# Temporal and spatial variability of prehistoric aquatic resource procurement: a case study from Mesolithic Northern Iberia

**DOI:** 10.1038/s41598-022-07239-8

**Published:** 2022-02-24

**Authors:** Stefania Milano, Bernd R. Schöne, Manuel R. González-Morales, Igor Gutiérrez-Zugasti

**Affiliations:** 1 Department of Human Evolution, Max Planck Institute for Evolutionary Anthropology, Leipzig, Germany; 2 Department of Evolutionary Ecology, Leibniz Institute for Zoo and Wildlife Research, Berlin, Germany; 3 Institute of Geosciences, University of Mainz, Mainz, Germany; 4 Instituto Internacional de Investigaciones Prehistóricas de Cantabria, Universidad de Cantabria, Gobierno de Cantabria, Banco Santander), Santander, Spain

**Keywords:** Climate-change adaptation, Palaeoclimate, Biogeochemistry

## Abstract

Prehistoric shell middens hold valuable evidence of past human–environment interactions. In this study, we used carbon (δ^13^C) and oxygen (δ^18^O) stable isotopes of *Mytilus galloprovincialis* shells excavated from El Perro, La Fragua and La Chora, three Mesolithic middens in Cantabria, Northern Spain, to examine hunter-gatherer subsistence strategies in terms of seasonality and collection areas. Furthermore, we used shell δ^18^O to reconstruct water temperature during the early Holocene. Stable isotopes reveal a shellfish harvesting diversification trend represented by the gradual establishment of the upper estuaries as new procurement areas and an increase of harvesting mobility in both coastal and in-land sites. These innovations in subsistence strategies during the Mesolithic coincided with major changes in the surrounding environment as attested by the water temperature reconstructions based on δ^18^O and backed by several global and regional records. Overall, our results show that shell δ^13^C and δ^18^O stable isotopes have an underexplored potential as provenance proxies which stimulates their application to the archaeological record to further understand prehistoric human resource procurement and diet.

## Introduction

Humans have been gathering shellfish for more than 150,000 years^1^. Mainly used as food resources, personal ornaments and tools, shell remains were accumulated in large quantities, and numerous ‘shell middens’ started to appear worldwide. Especially from the Middle Palaeolithic onwards, hunter-gatherer populations have increased their reliance on coastal environments, with a gradually more systematic use of marine resources^2–4^. These coastal adaptations represent a major change in the behavioural evolution of different hominin species and, besides the increase in dietary breadth, they involve settlement expansion (e.g. on offshore islands) and specific technological development (e.g. fishing nets, boats and other specialized maritime tools)^5^. In Atlantic Europe, the last hunter-fisher-gatherer societies of the Mesolithic, largely relied on marine resources, generating the usually called ‘shell middens’, a type of site where shells are the dominant component, although remains from other aquatic and terrestrial food resources and human activities are also present. The Mesolithic was a period of great geographical variability and high sociocultural diversity, but in general, coastal societies were characterized by diversified diets and reduced regional mobility^6^.

Marine organisms, although poor in caloric intake, are enriched in ‘brain-selective nutrients’, critical compounds for the development and maintenance of brain tissues^7^. For this reason, it has been argued the diversified diet associated to the coastal environments might have been related to an increase of individual fitness, cognitive and cultural complexity as well as population growth^8^. For decades, the importance of coasts as prehistoric settlement locations and migratory corridors has been underestimated but recent studies have confirmed these habitats played a crucial role in the evolutionary trajectories of human species^9–12^. Although methodological advancements brought interest to this subject, coastal human–environment interactions are still not fully elucidated.

Archaeological mollusc remains offer a unique opportunity to fill this knowledge gap, although a wide range of zooarchaeological remains (e.g. including terrestrial species) needs to be studied to obtain a comprehensive knowledge on composition of ancient human diets^13,14^. Due to their exceptional material properties, similar to aquatic vertebrate hard tissues, shells tend to survive in the archaeological contexts, allowing detailed analyses of their use by human populations through time^15,16^. Furthermore, the geochemical composition of the shell carbonates (aragonite and/or calcite) can be used to infer the environmental conditions occurring at the time of mollusc growth, which in turn is useful to understand past settlement patterns^17,18^. This information is derived from the shell δ^18^O, whose incorporation mainly depends on the surrounding sea water temperatures (SSTs) occurring at the time of deposition, allowing δ^18^O to be an accurate sub-seasonal palaeothermometer^19,20^. According to the last temperature recorded by the mollusc in the shell ventral margin, it is possible to seasonally contextualize the collection event^18,21^.

**Figure 1:**
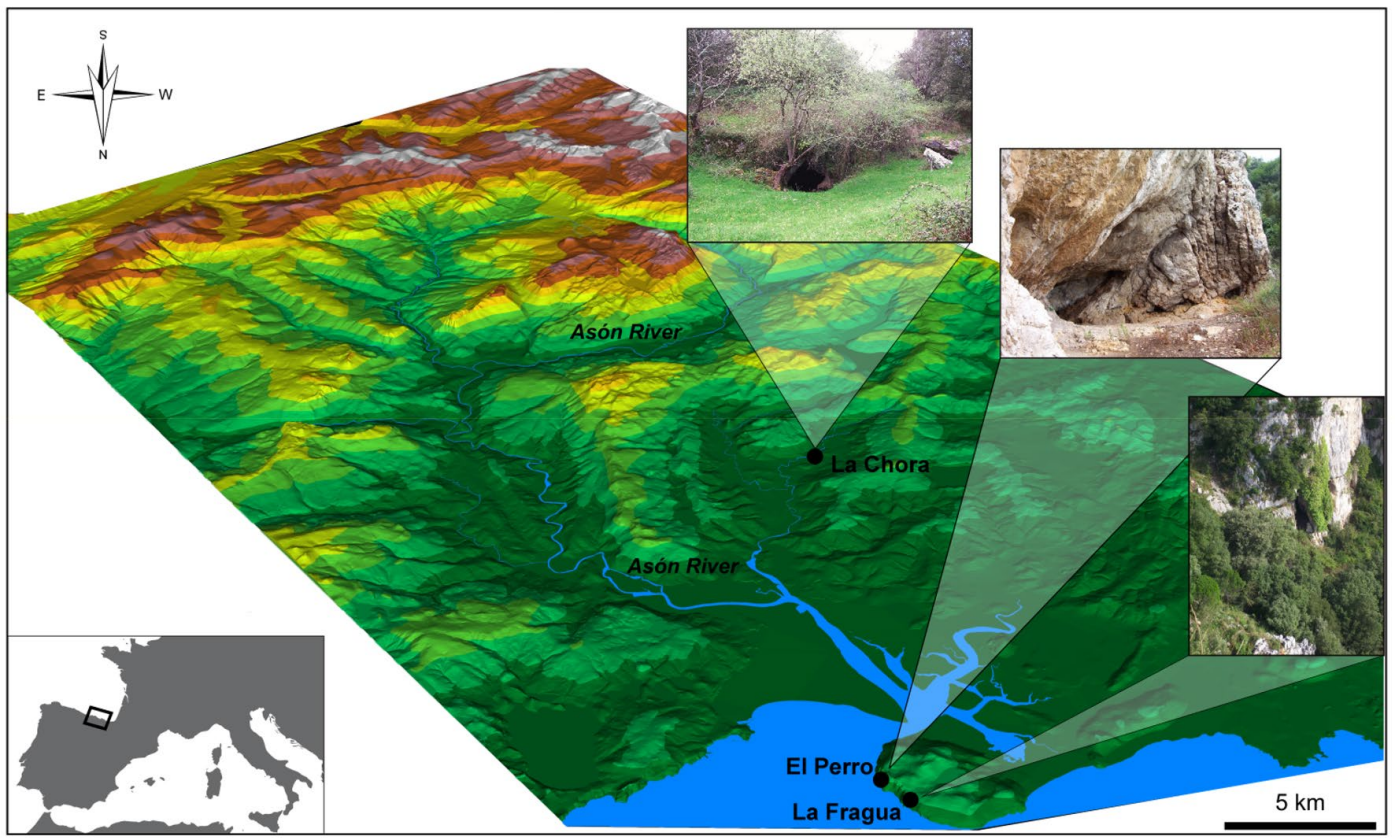
Location of the three studied archaeological shell middens (El Perro, La Fragua and La Chora) in the Asón River basin, in Cantabria, Northern Spain. Map produced using ArcMap 10.6 (ESRI, https://www.esri.com/en-us/arcgis/products/arcgis-desktop/resources) using the DTM25 cartography as base for the Digital Terrain Model (DTM), scale 1:25,000 with ETRS89 reference system of the National Geographic Institute of Spain.

In this study, the geochemical compositions of *Mytilus galloprovincialis* Lamarck, 1819 (Mediterranean mussel) shells excavated from the Mesolithic middens of El Perro, La Fragua and La Chora in Northern Spain (Fig. 1) were used to reconstruct collection patterns and local water palaeotemperatures. Previous studies focused on the δ^18^O variability as tool to investigate shellfish collection habitats^22^. However, the present work represents the first attempt to use both oxygen and carbon stable isotopes to understand prehistoric marine resource exploitation on both temporal spatial scale. Based on previous results on modern specimens^23^, the correlation between δ^18^O and carbon stable isotopes (δ^13^C) was used to discriminate the provenance of the shells between open coast (r = − 0.27), lower (r = − 0.13) and upper estuarine habitats (r = 0.56). In all, the proposed approach has the potential to expand the use of existing proxies to further interpret prehistoric hunter-gatherer relationships with their surroundings, throwing light on key aspects of the evolution of our species’ foraging practises.

## Results

### Radiocarbon dating (^14^C)

The dates obtained by the radiocarbon analysis on two *M. galloprovincialis* from El Perro (level 1.5) indicated an age range between 11,340 and 10,650 cal BP (2-σ error; Table S1). The results are in agreement with the *Patella vulgata* data from level 1.4 (11,170–10,560 cal BP 2-σ error; Table S1). The two specimens from La Fragua (levels 1.1 and 1.4) indicated an age range between 8250 and 7700 cal BP (2-σ error; Table S1). The two *M. galloprovincialis* from La Chora (level 103 and 104) indicated an age range between 8170 and 7800 cal BP (2-σ error; Table S1).

### Harvesting location and seasonality

Variability of the δ^13^C–δ^18^O correlation coefficients was observed in all three archaeological sites (Table S2). Based on the similarity with their modern counterpart (Fig. S1), each shell was assigned to a specific provenance habitat. The 95% confidence intervals (CI) of the correlation coefficients of modern specimens from fully marine (− 0.41 ≤ r ≤ − 0.12), lower estuarine (− 0.08 ≤ r ≤ 0.33) and upper estuarine habitats (0.40 ≤ r ≤ 0.69) were used as reference for the classification (Fig. S1). Nine shells showed extremely negative and positive correlations, with r values outside the CI limits. Presumably secondary environmental conditions occurring during shell deposition and entangled in the geochemical carbonate composition could have triggered such values. However, even if these variables could not be identified, correlations with r < − 0.41 and r > 0.69 were respectively assigned to open coast and upper estuary based on the negative-to-positive correlation gradient observed in our calibration study^23^.

**Figure 2:**
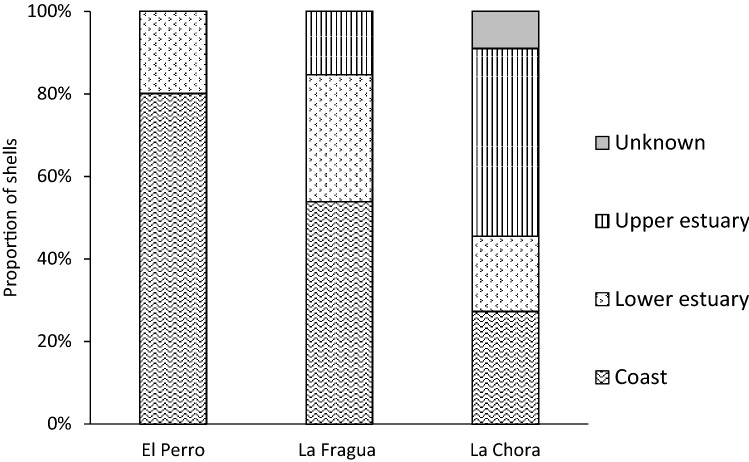
Provenance of the archaeological shells reconstructed using the oxygen and carbon stable isotope correlation obtained by sequential shell sub-sampling.

**Figure 3:**
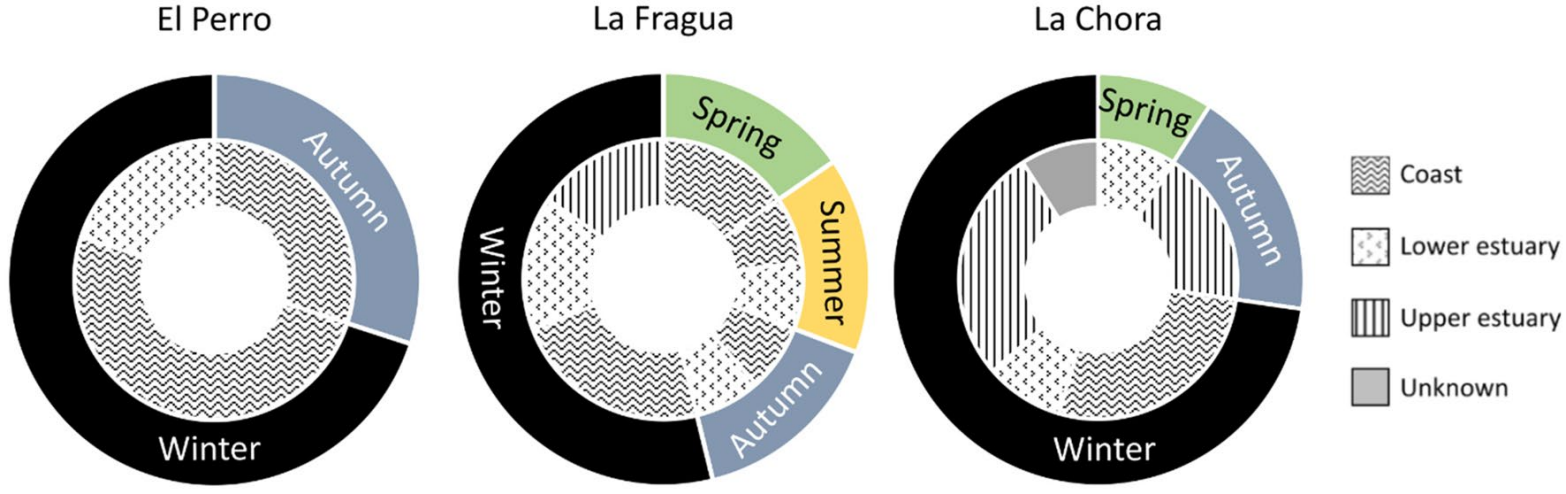
*M. galloprovincialis* harvesting seasonality and location in the three archaeological sites.

Eight out of the 10 shells excavated from El Perro (80%) were assigned to the open coast and two were assigned to the lower estuary (20%; Fig. 2). Among the 13 shells excavated from La Fragua, seven (54%) were assigned to the open coast, four (31%) were assigned to the lower estuary and two (15%) were assigned to the upper estuary (Fig. 2). Among the 11 shells excavated from La Chora, three (27%) were assigned to the open coast, two (18%) were assigned to the lower estuary, five (45%) were assigned to the upper estuary and one was of unknown location (Fig. 2). Classification to unknown harvesting location for shell 1688 resulted from the specific correlation coefficient (r = − 0.11) laying between the coastal and lower estuary confidence intervals. Therefore, its provenance could not be robustly defined. By using the archaeological shells with longer isotope sequences as baseline at each site and the quartile method, the season of harvesting was defined (Table S2). All the shells from El Perro (Fig. 3) were harvested during autumn (30%; n = 3) and winter (70%; n = 7). At La Fragua, the collection happened during different seasons: 54% of the shells (n = 7) were harvested during winter and 15% (n = 2) during each of the other three seasons (Fig. 3). The shells from La Chora were collected mostly in winter (82%, n = 8) but to a lesser degree also during autumn (18%, n = 2) and spring (9%; n = 1). When using only shells harvested from the open coast as baseline and excluding the estuarine specimens, seasonality results do not vary with one exception at La Chora (shell 1491) which would appear harvested in winter instead of autumn.

**Figure 4:**
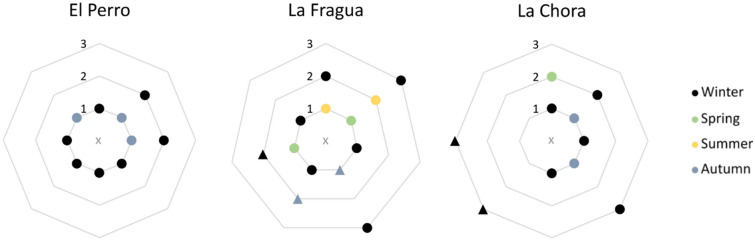
Delineative collection radius at the studied archaeological sites. The centre of each graph is the location of the shell midden. The concentric lines represent the different collection areas with a relative measure of distance from the site itself, from 1 (closest) to 3 (farthest). In El Perro and La Fragua 1 = open coast, 2 = lower estuary, 3 = upper estuary. In La Chora: 1 = upper estuary, 2 = lower estuary, 3 = open coast. The triangles indicate shells excavated from different units within the site (i.e. 1.4 in La Fragua and 104 in La Chora).

By combining provenance and collection seasonality results, our data show that at El Perro the mussels harvested at the open coast were collected mostly in winter (63%) and autumn (38%) and those from the lower estuary were all collected in winter (Fig. 3). Overall, the collection occurred in a relatively defined radius from the site (Fig. 4). At La Fragua instead, the radius of collection increased including all procurement areas (Fig. 4). Mussels from the open coast were collected mainly in winter (60%) and at a lesser degree during the other three seasons (40% in spring and 20% in summer and autumn; Fig. 3). The one coastal specimen collected in autumn belonged to unit 1.4, representing therefore a different collection event than the rest of the shells, from unit 1.1 (Fig. 4). Likewise, in the lower estuarine habitat, shellfish gathering occurred mainly during winter and autumn (50% and 25%, respectively) but also in summer (25%). Here, half of the specimens, collected in autumn and winter, were retrieved from unit 1.4 (Fig. 4). Collection in the upper estuary was recorded only in winter (Fig. 3). At La Chora, all the open coast mussels were harvested during winter and most of them were retrieved from unit 104 (Figs. 3, 4). Half of the mussels from lower estuarine habitats were harvested in winter and half in spring. Also, the shells from the upper estuary were collected during autumn and winter (40% and 60%, respectively).

### Palaeotemperature reconstruction

**Figure 5:**
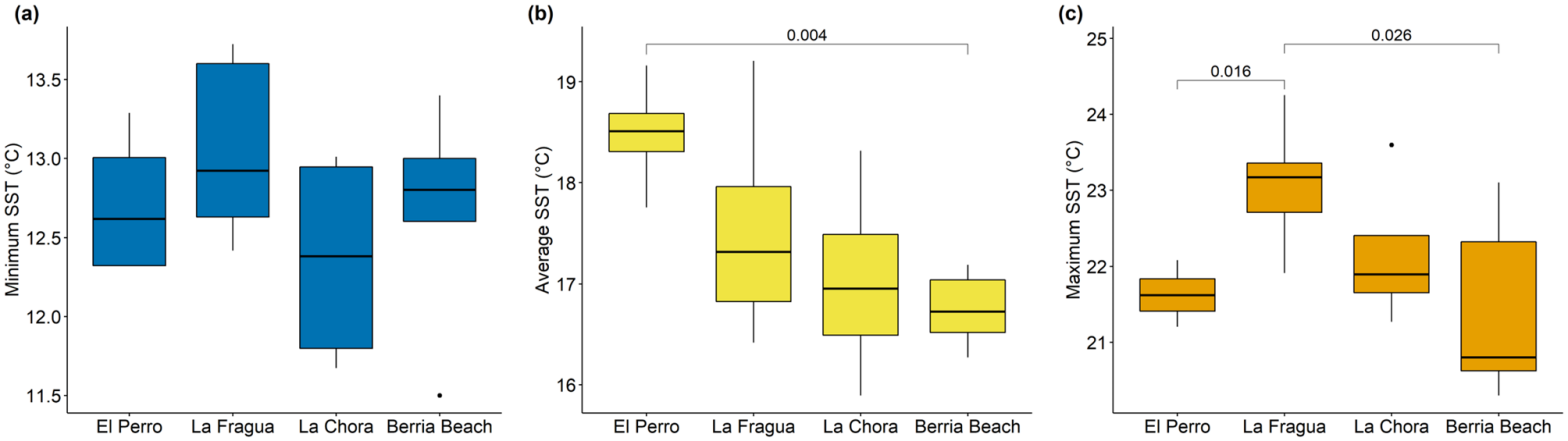
Sea surface temperature (SST) reconstruction using *M. galloprovincialis* shells from the three archaeological sites using δ^18^O_s_ as palaeothermometer. Reconstructed (**a**) minimum, (**b**) average and (**c**) maximum water temperatures. Shells collected from Berria Beach and previously analysed by Milano et al.^23^ were used as modern comparison. Significant differences (p < 0.05) are given in the graphs. Black circles identify the outliers, data points laying outside 1.5 times the interquartile range (defined by box whiskers).

The SSTs recorded by the *M. galloprovincialis* specimens from El Perro ranged from 12.7 ± 0.5 to 21.6 ± 0.4 °C, with an average of 18.5 ± 0.6 °C (Fig. 5). When only shells harvested from the open coast were used in the calculation, the SSTs were ranging from 12.6 ± 0.6 to 21.8 ± 0.3 °C, with an average of 18.7 ± 0.4 °C. At La Fragua, the SSTs ranged from 13.1 ± 0.6 to 23.1 ± 0.9 °C, with an average of 17.5 ± 1.1 °C (Fig. 5). When only shells harvested from the open coast were used in the calculation, the SSTs were ranging from 13.3 ± 0.6 to 23.4 ± 0.8 °C, with an average of 18.2 ± 1.0 °C. The shells from La Chora recorded SSTs ranging from 12.4 ± 0.8 to 22.2 ± 1.0 °C, with an average of 17.0 ± 1.0 °C (Fig. 5). When only specimens harvested from the open coast were used in the calculation, the SSTs were ranging from 12.4 ± 0.8 to 22.8 ± 1.1 °C, with an average of 17.8 ± 0.8 °C. As modern reference, specimens collected from Berria Beach and previously analysed by Milano et al. (2020) showed a SST range between 12.7 ± 0.7 and 21.4 ± 1.2 °C, with an average of 16.8 ± 0.4 °C (Fig. 5)^23^. The SST reconstructions using as baseline specimens from all habitats were not statistically different than the reconstructions based on open coast specimens only (t-test, p = 0.87).

A statistical difference was found between the average SST in El Perro and Berria Beach (p = 0.004; Fig. 5b). Furthermore, statistical differences were also found between the maximum SSTs in El Perro and La Fragua (p = 0.016) and between La Fragua and Berria Beach (p = 0.026; Fig. 5c).

## Discussion

The territory of northern Spain contains a very rich Upper Palaeolithic and Mesolithic archaeological record^24,25^. Coastal resources have been identified in many Upper Palaeolithic cave sites such as the renowned site of Altamira^26,27^. However, the formation of huge shell middens is mostly a Mesolithic phenomenon linked to a process of intensification in the use of coastal resources^28–30^. The three Cantabrian shell middens considered in this study are chronologically placed during the Early Holocene. Their detailed descriptions are given in the Supplementary Information (Figs. S2–S7, Tables S3–S5). El Perro and La Fragua were occupied during two different time windows: from ca. 11,340 to 10,650 cal BP (El Perro), corresponding to the Azilian-Mesolithic transition in the region (although Level 1 from El Perro has been culturally assigned to the Mesolithic) and from ca. 8250 to 7700 cal BP (La Fragua), corresponding to a more advanced phase of the Mesolithic^31^. These sites are ca. 800 m from each other and today they are situated along the coastline, but during their respective occupations El Perro was about 6–7 km and La Fragua about 0.5–1.5 km away from the coastline^32^. In fact, in good agreement with the Early Holocene global warming trend, the Cantabrian coastline between 12,000 and 7,800 cal BP was subjected to different transgression and regression events associated with a sea level rise from − 50 to − 7 m^33,34^. Differently, the records of La Fragua and La Chora encompass the same time period in two spatially different localities, ca. 12 km away from each other, with La Chora being the most in-land site.

Our results indicate that shell collection patterns changed considerably among the two different time intervals and the three locations. This observation is in line with a general trend detectable in all the Cantabrian region. During Late Magdalenian and Azilian (16,300–10,800 cal BP) coastal diet appeared largely specialized with few very abundant shellfish species such as the limpet *Patella vulgata* and the periwinkle *Littorina littorea.* Whereas bivalves, such as mussels and oysters, displayed an extremely limited contribution. However, during the Mesolithic (10,800–6,800 cal BP), the exploitation pattern changed dramatically and it became more diversified^28,35,36^. In our study, such diversification was especially perceptible in terms of procurement areas and collection radius. The shells from El Perro were mostly collected along the open coast, with some exceptions collected in the lower estuary. These two areas might have been the closest and most easily accessible from El Perro. At La Fragua, instead, for the first time the collection also occurred in the upper estuary, which likely represented the most distant habitat from the site. Similarly, at La Chora, some mussels were harvested from the nearby upper estuary but both lower estuary and open coast were used too. Previous studies on Mesolithic shell midden sites in the region have identified a radius of shell collection of 4–5 km from the sites^37,38^. Although the exact coastline configuration during the early Holocene is not well known, our data indicate that the radius may have been larger than that, taking into account the modern and estimated distances of the sites from the collection areas.

Furthermore, at El Perro, shellfish harvesting occurred exclusively during autumn and winter. This temporal selectivity in the resource procurement was previously observed for gastropod species in other nearby shell middens^39,40^. In this geographical context, it has been previously hypothesized that the use of coastal settlements was strictly seasonal and limited to the cold periods^39,41^. However, other researchers suggested that these sites were potentially also inhabited during the warmer phases based on the evidence of hunting at late spring and early summer^42^. In that case, the temporally constrained consumption of shellfish may be related to the availability of other resources comprised in the hunter-gatherer diets^43^. Our data showed that, during the older collection event in La Fragua (unit 1.4) the shells were collected in autumn and winter, similarly to El Perro. However, during the more recent event (unit 1.1) they were collected in winter, spring and summer. In La Chora, the older collection event (unit 104) was characterized by only winter collection, whereas spring and autumn were identified in the later collection event. Therefore, irrespective of the site location, our data showed that, although autumn and winter remain the preferred harvesting periods, at La Fragua and La Chora there was evidence of collection during warmer periods too.

When combining both temporal and spatial perspectives, our data revealed that at El Perro the collection was limited to the open coast and lower estuary areas and to the autumn–winter months. The same applies to the earliest collection event in La Fragua too. However, in the more recent collection event, the harvesting mobility during winter increased, including the upper estuary area. On the other hand, the evidence of mussel harvesting during warmer periods is spatially associated to the nearby open coast and lower estuary. At La Chora, the mobility was generally high, but harvesting was mainly constrained to autumn and winter.

The observed exploitation of the upper estuaries as new procurement areas coincided with drastic environmental changes emerged as consequences of the overall climate warming trend^44^. Although minimum and average SSTs were similar, increasing maximum temperatures were observed in our stable isotope reconstructions, in particular between El Perro and La Fragua. Although the intervals of the radiocarbon dates indicate that La Fragua and La Chora might have been occupied during the 8.2 ka event, the reconstructed temperatures suggested that they might have been occupied after the cold event. In general, this trend is in agreement with previous reconstructions using sedimentary cores from the Bay of Biscay identifying the period between 8200 and 7400 cal BP as a warm anomaly possibly related to greater intensity of the North Atlantic Current, and in particular of the North Atlantic subpolar gyre^45,46^. Furthermore, changed atmospheric circulation induced increased precipitation triggering the replacement of tundra habitats with deciduous forests similar to modern ones^44,47^. Importantly, the analysis of foraminiferal assemblages showed the first appearance of the current estuaries around 8900 cal BP^48^. Although the sediment constant remodelling challenges the understanding of how riverine discharge rates developed and how the estuary and coastline formed over time^49,50^, our results indicate that local hunter-gatherers were relying on the newly-formed habitats for their subsistence. Evidence of estuarine shellfish species was also observed in other Late Mesolithic middens in Northern Iberia (e.g. Pico Ramos, Arenillas, La Trecha, and San Antonio), and Portugal (e.g. Cabeço da Amoreira, Cabeço da Arruda), confirming the increase in the estuarine exploitation^28,37,50,51^.

Overall, our data and interpretation suggest two main conclusions. First, across the studied period the estuarine habitats represented new areas for the local hunter-fisher-gatherer populations to explore and collect shellfish. As a consequence, a gradual change in mobility patterns, with displacements to more distant collection areas (upper estuary), is related to shellfish procurement occurred in La Fragua. For the in-land site of La Chora, mobility patterns were mainly related to movements toward the lower estuary and open coast, implying estimated displacements for dietary purposes of ca. 12–14 km from the site. The collection in the open coast from La Chora is also validated by the presence in the archaeological record of *Patella depressa*, *Phorcus lineatus* and specially *Patella ulyssiponensis*, all species inhabiting very exposed shores (unpublished data).

Secondly, mussel collection, in particular when it involved longer distance movements, was a quasi-exclusive autumn–winter phenomenon. Although we found evidence of shellfish harvesting during other periods, this was restricted to La Fragua second collection event and was spatially limited to the surrounding areas. This trend might be related to the higher meat yield of mussels in winter than other seasons, potentially triggering the targeting of wider areas for their collection. A study on the reproductive cycle of *M. galloprovincialis* in Northern Spain is in line with our hypothesis, showing that gametogenesis (when the gonads are larger, and the meat yield is at its best) occurs in winter, while spawning in spring and summer^52^. This behaviour of shellfish collection at the time of higher meat yields has been also recorded for the topshell *P. lineatus* in the Mesolithic of Northern Spain^53^.

Finally, our study has shown the potential of combined seasonality and provenance analysis in order to provide detailed information on the subsistence strategies related to the use of aquatic resources. In combination with further zooarchaeological data, this approach can be crucial in understanding settlement patterns and mobility of prehistoric populations.

## Methods

### Radiocarbon dating (^14^C)

To estimate the age of three studied sites, several shells were radiocarbon dated. Two *M. galloprovincialis* from El Perro (43.44044935660 N, − 3.43565684231 W; level 1.5) were dated and used for isotope analysis (PR_8 and PR_9). In addition, two *Patella vulgata* shells from level 1.4 were radiocarbon dated. Two *M. galloprovincialis* shells from La Fragua (43.44574848190 N, − 3.42729117685 W; FR_1.1_2_2_1 and FR_1.4_3_1) and two from La Chora (43.34660688440 N, − 3.50114274760 W; Ch_1548 and Ch_1688), were dated using radiocarbon and milled for water temperature reconstruction. The age calibration was obtained using the marine calibration curve Marine20 in Oxcal 4.4 with a ΔR of − 290 ± 25, which was recalculated following the method established by Soares et al.^54^.

### Shell preparation

A total of 34 archaeological *M. galloprovincialis* were selected from the Mesolithic shell middens of El Perro (Unit 1.5; n = 10), La Fragua (Units 1.1 and 1.4; n = 13) and La Chora (Units 103 and 104; n = 11). Along the axis of the maximum growth of each shell, a layer of JB KWIK epoxy resin was applied. After drying, from each shell, a section of about 2.5 mm was cut using an IsoMet Low Speed Precision Cutter and it was mounted on a microscope slide. Shell exposed surface was ground on Buehler silicon carbide papers of different grit size (600, 1200, 2500, 4000) and it was polished on a Buehler VerduTex cloth with a 3 μm diamond suspension. A total of 622 carbonate samples were micromilled using a 300 µm-diameter conical bit (Komet/Gebr. Brasseler GmbH & Co. KG, model no. H52 104 003) mounted on a Rexim Minimo drill. At each site, four shells were sampled for water temperature reconstruction by microdrilling about 30 sequential calcite samples along the growth axis (Table S1). The rest of the shells were sampled for collection seasonality by microdrilling about 10 sequential calcite samples from each specimen (Table S1). The average distance between the individual craters was about 300 µm.

### Stable isotope analyses

The stable isotope analyses of the samples from El Perro and La Fragua (n = 432) were conducted at the University of Mainz using a ThermoFisher MAT 253 gas source isotope ratio mass spectrometer (CF-IRMS) coupled to a GasBench II. The carbonates were digested in He-flushed borosilicate exetainers at 72 °C using a water-free phosphoric acid and the released CO_2_ was measured against NBS-19 calibrated Carrara Marble (δ^13^C =  + 2.01 ‰; δ^18^O =  − 1.91 ‰) distributed by IVA Analysentechnik GmbH & Co. KG. The average precision error (1σ; computed from eight injections per sample) was better than 0.05‰ for δ^18^O and 0.03 ‰ for δ^13^C, and the long-term accuracy based on blindly measured NBS-19 samples (N = 421) was better than 0.04 ‰ for δ^18^O and 0.03 ‰ for δ^13^C. The stable isotope analyses of the samples from La Chora (n = 190) were conducted at the Department of Human Evolution in the Max Planck Institute for Evolutionary Anthropology. Here, the carbonates were digested at 70 °C using a water-free phosphoric acid and a Kiel IV automated carbonate preparation device and the CO_2_ was measured with a ThermoFisher MAT 253 Plus CF-IRMS. The carbonate data were calibrated against an IAEA-603 calibrated Carrara marble (δ^13^C =  + 1.87 ‰; δ^18^O =  − 1.64 ‰). Here, the average precision error (1σ; computed from eight measurements per sample) was better than 0.05‰ for δ^18^O and 0.03 ‰ for δ^13^C, and the long-term accuracy based on blindly measured IAEA-603 samples (N = 268) was better than 0.05 ‰ for δ^18^O and 0.03 ‰ for δ^13^C. All carbonate results were reported in per mil (‰) relative to the Vienna Pee-Dee Belemnite (VPDB) standard.

### Provenance, palaeotemperature and collection seasonality statistics

Shellfish provenance was determined by examining the relationship between δ^13^C and δ^18^O of the sequentially drilled carbonate samples from each shell. The strength and direction of the δ^13^C–δ^18^O relationships of the archaeological carbonates were compared to the reference dataset based on modern mussels and published by Milano et al.^23^. In this study, correlations were shown to have a geographical gradient from negative in shells grown in fully marine conditions (r = − 0.27) to positive in upper estuarine habitats (r = 0.56) Shells from the lower estuarine area showed weak correlations (r = 0.13). Here, Fisher z-transformations were used to calculate the 95%confidence intervals for each of the three modern correlation coefficients. Subsequently, the harvesting location of the archaeological shells was determined based on which confidence interval their coefficients would lie within (Fig. S1). At each site, the harvesting season was determined using as a baseline the δ^18^O range of the archaeological shells subsampled for SST reconstruction, which was divided into quartiles. Each quartile represented one of the four seasons. According to which quartile the shell edge δ^18^O values would fall in, the season of collection was determined. Furthermore, for each shell, the isotope trend of the last ten data points preceding the shell edge was used to confirm the season assignment.

For the SST reconstruction, shell δ^18^O values were converted into water temperatures using calcite fractionation equations by Friedman and O’Neil (1977):

1 $${1}000{\text{ln}}\alpha = \left( {{2}.{78} \times {1}0^{{6}} /{\text{T}}^{{2}} } \right){-}{2}.{89}$$

2 $$\alpha = \left( {{1}000 + \delta^{{{18}}} {\text{O}}_{{{\text{s }}({\text{SMOW}})}} } \right)/\left( {{1}000 + \delta^{{{18}}} {\text{O}}_{{{\text{w }}({\text{SMOW}})}} } \right)$$

where α is the water-calcite fractionation factor and T is the temperature recorded (expressed in Kelvin) and δ^18^O_w_ is the isotopic ratio of the water. The archaeological δ^18^O_w_ was estimated starting from the modern value (0.9‰). The estimation implied a further calculation of the sea level at the time of the site occupation^32^ and a multiplication by a specific correction factor (0.011 ‰)^55^. Minimum, average and maximum SSTs were computed as averages (and standard deviations) of the respective values measured in the single shells within each site. ANOVA followed by pairwise t-tests were used to assess potential SST changes through time. All the statistical analyses were executed using the R 4.1.2 software (https://www.R-project.org/)^56^.

## Supplementary Information

Supplementary Information.
