# Supplementary material for: Temporal and spatial variability of prehistoric aquatic resource procurement: a case study from Mesolithic Northern Iberia
Source: Sci Rep. 2022 Feb 24;12:3111. doi: 10.1038/s41598-022-07239-8 (PMC8873316; doi:10.1038/s41598-022-07239-8)
Supplement: Supplementary file 1 — Supplementary Information. [file 41598_2022_7239_MOESM1_ESM.docx]

Table S1. Details of the archaeological shells used in this study. The age established by direct radiocarbon dating was calculated using a 2-σ error range.

| **Site** | **Unit** | **Shell ID** | **SST reconstruction** | **Provenance** | **Collection seasonality** | **Age (BP)** | **Age (cal BP 2σ)** | **Dating lab reference** | **Sea level (m)** |
| --- | --- | --- | --- | --- | --- | --- | --- | --- | --- |
| El Perro | 1.4 | *P. vulgata* |  |  |  | 9,660 ± 45 | 11,059 - 10,588 | ﻿OxA-33179 | -38 |
|  | 1.4 | *P. vulgata* |  |  |  | 9,780 ± 40 | 11,173 - 10,758 | OxA-34388 | -38 |
|  | 1.4 | *P. vulgata* |  |  |  | 9,770 ± 40 | 11,165 - 10,747 | OxA-34389 | -38 |
|  | 1.5 | PR_1 |  | x | x |  |  |  |  |
|  | 1.5 | PR_2 |  | x | x |  |  |  |  |
|  | 1.5 | PR_3 |  | x | x |  |  |  |  |
|  | 1.5 | PR_4 | x | x | x |  |  |  |  |
|  | 1.5 | PR_5 |  | x | x |  |  |  |  |
|  | 1.5 | PR_6 | x | x | x |  |  |  |  |
|  | 1.5 | PR_8 | x | x | x | 9,910 ± 40 | 11,340 - 10,953 | ICA-19S/10105 | -38 |
|  | 1.5 | PR_9 | x | x | x | 9,690 ± 30 | 11,074 - 10,653 | ICA-19S/10106 | -38 |
|  | 1.5 | PR_11 |  | x | x |  |  |  |  |
|  | 1.5 | PR_12 |  | x | x |  |  |  |  |
| La Fragua | 1.1 | FR_1.1_2.2.1 | x | x | x | 7,320 ± 40 | 8,030 - 7,710 | ICA-17S/0436 | -11 |
|  | 1.1 | FR_1.1_2.2.2 | x | x | x |  |  |  |  |
|  | 1.1 | FR_1.1_2.2.3 |  | x | x |  |  |  |  |
|  | 1.1 | FR_1.1_2.2.4 |  | x | x |  |  |  |  |
|  | 1.1 | FR_1.1_2.3.1 | x | x | x |  |  |  |  |
|  | 1.1 | FR_1.1_2.3.2 | x | x | x |  |  |  |  |
|  | 1.1 | FR_1.1_2.3.3 |  | x | x |  |  |  |  |
|  | 1.1 | FR_1.1_2.3.4 |  | x | x |  |  |  |  |
|  | 1.1 | FR_1.1_2.3.6 |  | x | x |  |  |  |  |
|  | 1.1 | FR_1.1_2.3.7 |  | x | x |  |  |  |  |
|  | 1.4 | FR_1.4_3.1 |  |  |  | 7,500 ± 40 | 8,249 - 7,916 | ICA-17S/0435 | -12 |
|  | 1.4 | FR_1.4_3.2 | x | x | x |  |  |  |  |
|  | 1.4 | FR_1.4_3.4 |  | x | x |  |  |  |  |
|  | 1.4 | FR_1.4_3.5 |  | x | x |  |  |  |  |
| La Chora | 103 | CH_1491 | x | x | x |  |  |  |  |
|  | 103 | CH_1503 |  | x | x |  |  |  |  |
|  | 103 | CH_1504 |  | x | x |  |  |  |  |
|  | 103 | CH_1506 |  | x | x |  |  |  |  |
|  | 103 | CH_1546 |  | x | x |  |  |  |  |
|  | 103 | CH_1547 | x | x | x |  |  |  |  |
|  | 103 | CH_1548 | x | x | x | 7,390 ± 40 | 8,136 - 7,796 | ICA-19S/0177 | -12 |
|  | 103 | CH_1563 |  | x | x |  |  |  |  |
|  | 103 | CH_1611 |  | x | x |  |  |  |  |
|  | 104 | CH_1687 |  | x | x |  |  |  |  |
|  | 104 | CH_1688 | x | x | x | 7,430 ± 40 | 8,166 - 7,845 | ICA-19S/0176 | -12 |

Table S2. Carbon and oxygen stable isotopes sequences and their relationships (Pearson’s r coefficient) in the archaeological shells analysed. Shells subsampled for SST reconstruction were used as baseline in the identification of the season of harvesting using a quartile method (minimum, Q1, Q2, Q3, maximum). Harvesting location and season are indicated for each specimen (L Estuary = lower estuary, U Estuary = upper estuary).

| **El Perro** | | | | | | | | | | | | | | | | | | | | | | | | | | |
| --- | --- | --- | --- | --- | --- | --- | --- | --- | --- | --- | --- | --- | --- | --- | --- | --- | --- | --- | --- | --- | --- | --- | --- | --- | --- | --- |
|  | **PR 1** | | **PR 2** | | **PR 3** | | **PR 4** | | **PR 5** | | **PR 6** | | **PR 8** | | **PR 9** | | **PR 11** | | **PR 12** | |  |  |  |  |  |  |
|  | **δ^13^C_s_** | **δ^18^O_s_** | **δ^13^C_s_** | **δ^18^O_s_** | **δ^13^C_s_** | **δ^18^O_s_** | **δ^13^C_s_** | **δ^18^O_s_** | **δ^13^C_s_** | **δ^18^O_s_** | **δ^13^C_s_** | **δ^18^O_s_** | **δ^13^C_s_** | **δ^18^O_s_** | **δ^13^C_s_** | **δ^18^O_s_** | **δ^13^C_s_** | **δ^18^O_s_** | **δ^13^C_s_** | **δ^18^O_s_** |  |  |  |  |  |  |
| Shell edge | *0.22* | *1.73* | *0.1* | *2.17* | *-0.60* | *1.47* | *-1.13* | *1.96* | *-0.05* | *1.45* | *-0.83* | *1.87* | *-0.84* | *2.10* | *-0.33* | *2.10* | *-0.45* | *1.72* | *-1.28* | *1.07* |  |  |  |  |  |  |
|  | 0.33 | 1.15 | 0.09 | 0.98 | -0.24 | 1.40 | -0.23 | 1.10 | -0.66 | 1.38 | -0.31 | 1.31 | 0.06 | 0.80 | -0.52 | 1.24 | -0.56 | 1.33 | -1.01 | 1.06 |  |  |  |  |  |  |
|  | 0.48 | 0.62 | -0.37 | 0.86 | -0.17 | 1.26 | -0.12 | 0.60 | -0.49 | 1.47 | -0.19 | 0.70 | 0.30 | 0.43 | -0.69 | 0.81 | -0.55 | 1.38 | -0.67 | 0.67 |  |  |  |  |  |  |
|  | 0.61 | 0.59 | -0.68 | 0.96 | -0.37 | 1.10 | -0.43 | 0.50 | -0.42 | 1.26 | -0.54 | 0.31 | 0.38 | 0.30 | -0.19 | 0.79 | -0.75 | 1.58 | -0.54 | 0.62 |  |  |  |  |  |  |
|  | 0.79 | 0.73 | -0.35 | 1.73 | -0.29 | 0.85 | -0.84 | 1.19 | 0.53 | 0.87 | -0.77 | 0.18 | 0.04 | 0.34 | -0.40 | 0.40 | -0.46 | 1.60 | -0.62 | 0.62 |  |  |  |  |  |  |
|  | 0.56 | 1.04 | 0.23 | 1.73 | -0.43 | 0.62 | -0.20 | 1.30 | 0.15 | 0.59 | -0.81 | 0.01 | -0.42 | 1.18 | -0.28 | 0.75 | 0.09 | 1.73 | -0.72 | 1.12 |  |  |  |  |  |  |
|  | 0.26 | 1.58 | 0.24 | 1.46 | 0.01 | 0.82 | -0.36 | 0.48 | -0.09 | 0.06 | -0.54 | 0.24 | 0.22 | 0.92 | -0.29 | 1.58 | 0.10 | 1.24 | -0.27 | 0.83 |  |  |  |  |  |  |
|  | 0.47 | 1.90 | 0.31 | 1.11 | 0.20 | 1.14 | -0.46 | 0.33 | 0.51 | 0.17 | -0.83 | 1.11 | 0.15 | 0.16 | -0.08 | 1.32 | 0.47 | 1.06 | 0.37 | 0.42 |  |  |  |  |  |  |
|  | 0.47 | 1.79 | 0.17 | 0.68 | 0.05 | 0.94 | -0.41 | 0.86 |  |  | -0.42 | 1.09 | -0.24 | 0.09 | 0.40 | 1.01 | 0.65 | 0.91 | 0.18 | 0.28 |  |  |  |  |  |  |
|  | 0.43 | 0.77 | 0.01 | 0.56 | 0.04 | 0.58 | -0.11 | 0.42 |  |  | -0.19 | 0.88 | -0.76 | 0.42 | 0.52 | 0.57 | 0.60 | 0.75 | 0.02 | 0.44 |  |  |  |  |  |  |
|  | -0.16 | 1.04 | 0.52 | 1.04 | -0.11 | 0.56 | -0.13 | 0.34 |  |  | -0.08 | 0.78 | -1.02 | 1.01 | 0.54 | 0.25 |  |  | -0.45 | 0.62 |  |  |  |  |  |  |
|  |  |  |  |  |  |  | -0.30 | 0.80 |  |  | -0.04 | 0.61 | -0.65 | 0.83 | 0.33 | 0.09 |  |  |  |  |  |  |  |  |  |  |
|  |  |  |  |  |  |  | -0.43 | 1.29 |  |  | -0.47 | 0.36 | -0.32 | 0.38 | 0.15 | 0.06 |  |  |  |  |  |  |  |  |  |  |
|  |  |  |  |  |  |  | -0.13 | 1.46 |  |  | -0.30 | 0.23 | -0.42 | 0.39 | 0.09 | -0.05 |  |  |  |  |  |  |  |  |  |  |
|  |  |  |  |  |  |  | 0.21 | 1.79 |  |  | -0.25 | 0.20 | -0.26 | 0.23 | 0.17 | 0.10 |  |  |  |  |  |  |  |  |  |  |
|  |  |  |  |  |  |  | -0.25 | 1.47 |  |  | -0.43 | 0.15 | -0.41 | 0.15 | 0.03 | 0.30 |  |  |  |  |  |  |  |  |  |  |
|  |  |  |  |  |  |  | 0.24 | 0.80 |  |  | -0.32 | 0.22 | -0.36 | -0.12 | -0.43 | 0.31 |  |  |  |  |  |  |  |  |  |  |
|  |  |  |  |  |  |  | 0.01 | 0.51 |  |  | -0.24 | 0.10 | -0.38 | 0.09 | -0.54 | 0.54 |  |  |  |  |  |  |  |  |  |  |
|  |  |  |  |  |  |  | 0.03 | 1.05 |  |  | -0.03 | 0.16 | -0.24 | 0.07 | -0.44 | 0.71 |  |  |  |  |  |  |  |  |  |  |
|  |  |  |  |  |  |  | 0.04 | 1.48 |  |  | -0.06 | 0.18 | -0.35 | 0.19 | -0.73 | 1.27 |  |  |  |  |  |  |  |  |  |  |
|  |  |  |  |  |  |  | 0.01 | 1.33 |  |  | -0.06 | 0.35 | -0.40 | 0.48 | -0.46 | 1.36 |  |  |  |  |  |  |  |  |  |  |
|  |  |  |  |  |  |  | -0.11 | 0.99 |  |  | -0.38 | 0.48 | -0.50 | 0.55 | 0.13 | 1.37 |  |  |  |  |  |  |  |  |  |  |
|  |  |  |  |  |  |  | -0.19 | 0.74 |  |  | -0.53 | 0.56 | -0.81 | 1.01 | -0.11 | 0.76 |  |  |  |  |  |  |  |  |  |  |
|  |  |  |  |  |  |  | -0.53 | 0.59 |  |  |  |  | -0.78 | 1.48 | 0.02 | 0.42 |  |  |  |  |  |  |  |  |  |  |
|  |  |  |  |  |  |  | -0.46 | 0.17 |  |  |  |  | -0.48 | 1.44 | 0.03 | 0.10 |  |  |  |  |  |  |  |  |  |  |
|  |  |  |  |  |  |  | -0.43 | 0.35 |  |  |  |  | -0.30 | 1.44 | -0.05 | 0.15 |  |  |  |  |  |  |  |  |  |  |
|  |  |  |  |  |  |  | -0.53 | 0.33 |  |  |  |  | 0.08 | 1.47 | -0.28 | 0.19 |  |  |  |  |  |  |  |  |  |  |
|  |  |  |  |  |  |  | -0.31 | 0.13 |  |  |  |  | 0.05 | 1.26 | -0.33 | 0.40 |  |  |  |  |  |  |  |  |  |  |
|  |  |  |  |  |  |  | -0.20 | 0.07 |  |  |  |  | -0.36 | 0.65 | -0.51 | 0.50 |  |  |  |  |  |  |  |  |  |  |
|  |  |  |  |  |  |  |  |  |  |  |  |  | -0.27 | 0.50 | -0.72 | 0.60 |  |  |  |  |  |  |  |  |  |  |
|  |  |  |  |  |  |  |  |  |  |  |  |  | -0.14 | 0.50 | -1.25 | 0.96 |  |  |  |  |  |  |  |  |  |  |
|  |  |  |  |  |  |  |  |  |  |  |  |  |  |  | -0.93 | 1.14 |  |  |  |  |  |  |  |  |  |  |
| Minimum |  |  |  |  |  |  |  | 0.07 |  |  |  | 0.01 |  | -0.12 |  | -0.05 |  |  |  |  |  |  |  |  |  |  |
| Q1 |  |  |  |  |  |  |  | 0.54 |  |  |  | 0.47 |  | 0.44 |  | 0.49 |  |  |  |  |  |  |  |  |  |  |
| Q2 |  |  |  |  |  |  |  | 1.25 |  |  |  | 1.17 |  | 1.27 |  | 1.29 |  |  |  |  |  |  |  |  |  |  |
| Q3 |  |  |  |  |  |  |  | 1.08 |  |  |  | 1.00 |  | 1.06 |  | 1.09 |  |  |  |  |  |  |  |  |  |  |
| Maximum |  |  |  |  |  |  |  | 1.96 |  |  |  | 1.87 |  | 2.10 |  | 2.10 |  |  |  |  |  |  |  |  |  |  |
| r | -0.30 | | 0.09 | | -0.32 | | 0.01 | | -0.62 | | -0.23 | | -0.29 | | -0.37 | | -0.75 | | -0.84 | |  |  |  |  |  |  |
| Provenance | Coast | | L Estuary | | Coast | | L Estuary | | Coast | | Coast | | Coast | | Coast | | Coast | | Coast | |  |  |  |  |  |  |
| Collection season | Winter | | Winter | | Autumn | | Winter | | Autumn | | Winter | | Winter | | Winter | | Winter | | Autumn | |  |  |  |  |  |  |
| **La Fragua** | | | | | | | | | | | | | | | | | | | | | | | | | | |
|  | **FR 1.1_2_2_1** | | **FR 1.1_2_2_2** | | **FR 1.1_2_2_3** | | **FR 1.1_2_2_4** | | **FR 1.1_2_3_1** | | **FR 1.1_2_3_2** | | **FR 1.1_2_3_3** | | **FR 1.1_2_3_4** | | **FR 1.1_2_3_6** | | **FR 1.1_2_3_7** | | **FR 1.4_3_2** | | **FR 1.4_3_4** | | **FR 1.4_3_5** | |
|  | **δ^13^C_s_** | **δ^18^O_s_** | **δ^13^C_s_** | **δ^18^O_s_** | **δ^13^C_s_** | **δ^18^O_s_** | **δ^13^C_s_** | **δ^18^O_s_** | **δ^13^C_s_** | **δ^18^O_s_** | **δ^13^C_s_** | **δ^18^O_s_** | **δ^13^C_s_** | **δ^18^O_s_** | **δ^13^C_s_** | **δ^18^O_s_** | **δ^13^C_s_** | **δ^18^O_s_** | **δ^13^C_s_** | **δ^18^O_s_** | **δ^13^C_s_** | **δ^18^O_s_** | **δ^13^C_s_** | **δ^18^O_s_** | **δ^13^C_s_** | **δ^18^O_s_** |
| Shell edge | *0.20* | *0.90* | *0.05* | *0.81* | *-1.79* | *1.48* | *-1.56* | *1.82* | *-0.16* | *-0.29* | *0.16* | *-0.48* | *1.17* | *1.37* | *-1.07* | *1.85* | *0.26* | *1.68* | *-0.59* | *1.63* | *0.25* | *0.41* | *-0.66* | *1.18* | *0.60* | *0.90* |
|  | 0.22 | 1.65 | 0.05 | 1.54 | 0.02 | 1.36 | -0.70 | 0.62 | 0.52 | 0.98 | 0.13 | -0.31 | 1.25 | 0.89 | 0.83 | 1.72 | 0.46 | -0.31 | -0.03 | 1.28 | -0.06 | 0.05 | -0.42 | 0.01 | 0.21 | 0.19 |
|  | 0.39 | 1.66 | 0.12 | 1.59 | 0.07 | 0.75 | -0.98 | 0.22 | 0.55 | 0.32 | 0.10 | -0.73 | 1.22 | 0.78 | 0.75 | 1.41 | 0.48 | -0.46 | 0.20 | 1.02 | -0.16 | -0.34 | -0.71 | 0.74 | -0.49 | -0.36 |
|  | 0.46 | 1.62 | 0.29 | 1.73 | 0.01 | 0.22 | -1.00 | 0.77 | 0.54 | -0.06 | 0.05 | -0.51 | 1.09 | 0.75 | 0.56 | 1.35 | 0.48 | -0.58 | 0.20 | 0.76 | -0.25 | -0.54 | -0.46 | 1.47 | -0.60 | 0.53 |
|  | 0.46 | 1.25 | 0.12 | 1.50 | -0.11 | -0.08 | -1.14 | 1.40 | 0.39 | -0.16 | 0.25 | -0.33 | 0.95 | 0.58 | 0.41 | 1.06 | 0.58 | -0.51 | 0.08 | 0.65 | -0.14 | -0.35 | 0.37 | 1.02 | 0.07 | 1.47 |
|  | 0.50 | 1.16 | 0.39 | 1.28 | -0.63 | 1.31 | -0.79 | 1.75 | 0.23 | 0.16 | 0.27 | 0.02 | 0.47 | -0.14 | 0.46 | 1.02 | 0.61 | -0.42 | -0.64 | 0.18 | -0.03 | -0.33 | 0.15 | 0.50 | 0.36 | 1.26 |
|  | 0.71 | 1.11 | 0.37 | 1.17 | -0.21 | 1.87 | -0.54 | 1.15 | 0.08 | 1.13 | -0.13 | 0.60 | 0.19 | -0.49 | 0.57 | 0.73 | 0.45 | -0.42 | -0.54 | 0.20 | 0.07 | -0.32 | -0.78 | -0.20 | -0.12 | -0.07 |
|  | 0.58 | 0.78 | 0.51 | 0.97 | 0.19 | 1.70 | -1.12 | 0.27 | 0.08 | 1.61 | -0.35 | 0.67 | 0.04 | -0.50 | 0.79 | 0.66 | 0.75 | -0.14 | -0.85 | -0.12 | -0.07 | -0.05 | -0.83 | -0.37 | -0.86 | 1.13 |
|  | 0.53 | 0.70 | 0.42 | 0.85 | 0.34 | 1.47 | -0.86 | 1.06 | 0.43 | 1.65 | -0.31 | 0.94 | 0.16 | -0.07 | 0.80 | 0.64 | 0.71 | 0.01 | -0.83 | -0.61 | -0.48 | 0.62 | -0.74 | 0.45 | -0.35 | 1.50 |
|  | 0.56 | 0.51 | 0.20 | 0.55 | 0.64 | 1.31 | -0.28 | 0.90 | 0.80 | 1.78 | -0.22 | 1.03 | 0.17 | 0.26 | 0.77 | 0.28 | 0.74 | 0.50 | -0.55 | -0.64 | -0.46 | 0.59 | -0.87 | 0.92 | 0.39 | 1.21 |
|  | 0.77 | 0.42 | 0.12 | 0.08 | -0.32 | -0.07 | -0.80 | 0.40 | 0.72 | 1.36 | -0.11 | 1.21 | 0.17 | 0.39 | 0.89 | 0.19 | 0.53 | 0.36 |  |  | -0.56 | 0.67 | -0.96 | 1.36 | 0.61 | 0.78 |
|  | 1.06 | 0.13 | 0.18 | -0.21 |  |  |  |  | 0.74 | 0.52 | -0.15 | 1.02 |  |  | 0.96 | -0.03 |  |  |  |  | -0.54 | 1.05 |  |  |  |  |
|  | 1.06 | -0.08 | 0.11 | -0.40 |  |  |  |  | 0.88 | 0.59 | 0.15 | 0.58 |  |  |  |  |  |  |  |  | -0.60 | 1.22 |  |  |  |  |
|  | 0.87 | -0.33 | -0.02 | -0.47 |  |  |  |  | 0.82 | 0.36 | 0.37 | 0.59 |  |  |  |  |  |  |  |  | -0.28 | 1.51 |  |  |  |  |
|  | 0.67 | -0.40 | 0.04 | -0.49 |  |  |  |  | 0.60 | -0.09 | 0.24 | 0.07 |  |  |  |  |  |  |  |  | -0.21 | 1.51 |  |  |  |  |
|  | 0.76 | -0.65 | 0.14 | -0.69 |  |  |  |  | 0.51 | -0.38 | 0.23 | -0.55 |  |  |  |  |  |  |  |  | -0.06 | 1.46 |  |  |  |  |
|  | 0.93 | -0.47 | 0.44 | -0.53 |  |  |  |  | 0.73 | 0.09 | 0.27 | -0.78 |  |  |  |  |  |  |  |  | 0.08 | 1.17 |  |  |  |  |
|  | 0.90 | -0.43 | 0.36 | -0.36 |  |  |  |  | 0.57 | 1.09 | 0.66 | -0.40 |  |  |  |  |  |  |  |  | 0.02 | 0.95 |  |  |  |  |
|  | 0.88 | -0.27 | 0.34 | -0.41 |  |  |  |  | 0.41 | 1.31 | -0.27 | 0.27 |  |  |  |  |  |  |  |  |  |  |  |  |  |  |
|  | 0.95 | 0.02 | 0.09 | -0.19 |  |  |  |  | 0.33 | 1.37 | 0.13 | 0.79 |  |  |  |  |  |  |  |  |  |  |  |  |  |  |
|  | 0.80 | 0.12 | 0.18 | -0.31 |  |  |  |  | 0.53 | 1.58 | 0.02 | 1.18 |  |  |  |  |  |  |  |  |  |  |  |  |  |  |
|  | 0.66 | 0.34 | 0.50 | -0.15 |  |  |  |  | 0.79 | 1.66 | 0.15 | 1.47 |  |  |  |  |  |  |  |  |  |  |  |  |  |  |
|  | 0.46 | 0.58 | 0.52 | -0.01 |  |  |  |  | 1.06 | 1.52 | 0.47 | 1.43 |  |  |  |  |  |  |  |  |  |  |  |  |  |  |
|  | 0.24 | 0.63 | 0.36 | 0.27 |  |  |  |  | 1.21 | 1.66 | 0.79 | 0.89 |  |  |  |  |  |  |  |  |  |  |  |  |  |  |
|  | 0.23 | 0.95 | 0.07 | 0.44 |  |  |  |  | 1.28 | 1.46 | 0.66 | 0.59 |  |  |  |  |  |  |  |  |  |  |  |  |  |  |
|  | 0.22 | 1.12 | 0.15 | 0.27 |  |  |  |  | 1.48 | 1.38 | 0.46 | 0.29 |  |  |  |  |  |  |  |  |  |  |  |  |  |  |
|  | 0.23 | 1.22 | 0.16 | 0.60 |  |  |  |  | 1.60 | 1.17 | 0.69 | 0.03 |  |  |  |  |  |  |  |  |  |  |  |  |  |  |
|  | 0.32 | 1.32 | 0.07 | 0.65 |  |  |  |  | 1.54 | 1.06 | 0.70 | -0.26 |  |  |  |  |  |  |  |  |  |  |  |  |  |  |
|  | -0.16 | 1.54 | 0.15 | 0.75 |  |  |  |  | 1.29 | -0.03 | 0.59 | -0.59 |  |  |  |  |  |  |  |  |  |  |  |  |  |  |
|  | -0.26 | 1.44 | 0.23 | 0.95 |  |  |  |  |  |  | 0.43 | -0.83 |  |  |  |  |  |  |  |  |  |  |  |  |  |  |
|  | -0.15 | 1.22 | 0.22 | 0.81 |  |  |  |  |  |  | 0.45 | -0.54 |  |  |  |  |  |  |  |  |  |  |  |  |  |  |
|  | 0.17 | 1.28 | 0.05 | 1.05 |  |  |  |  |  |  | 0.21 | -0.36 |  |  |  |  |  |  |  |  |  |  |  |  |  |  |
|  | 0.29 | 1.33 | -0.07 | 1.00 |  |  |  |  |  |  | 0.12 | -0.02 |  |  |  |  |  |  |  |  |  |  |  |  |  |  |
|  | 0.38 | 1.16 | 0.05 | 1.35 |  |  |  |  |  |  | -0.24 | 0.18 |  |  |  |  |  |  |  |  |  |  |  |  |  |  |
|  | 0.34 | 1.16 | 0.30 | 1.60 |  |  |  |  |  |  | -1.26 | -0.08 |  |  |  |  |  |  |  |  |  |  |  |  |  |  |
|  | 0.52 | 1.41 | 0.40 | 1.47 |  |  |  |  |  |  | -0.57 | 0.58 |  |  |  |  |  |  |  |  |  |  |  |  |  |  |
|  | 0.53 | 1.41 | 0.28 | 1.33 |  |  |  |  |  |  | -0.69 | 0.86 |  |  |  |  |  |  |  |  |  |  |  |  |  |  |
|  | 0.55 | 1.43 | -0.20 | 1.54 |  |  |  |  |  |  | -0.57 | 1.12 |  |  |  |  |  |  |  |  |  |  |  |  |  |  |
|  |  |  | -0.21 | 1.35 |  |  |  |  |  |  | -0.28 | 1.14 |  |  |  |  |  |  |  |  |  |  |  |  |  |  |
|  |  |  | -0.22 | 1.32 |  |  |  |  |  |  | -0.28 | 0.98 |  |  |  |  |  |  |  |  |  |  |  |  |  |  |
|  |  |  | -0.09 | 1.46 |  |  |  |  |  |  | -0.37 | 0.20 |  |  |  |  |  |  |  |  |  |  |  |  |  |  |
|  |  |  | -0.10 | 1.31 |  |  |  |  |  |  | 0.40 | 0.14 |  |  |  |  |  |  |  |  |  |  |  |  |  |  |
|  |  |  |  |  |  |  |  |  |  |  | 0.46 | -0.39 |  |  |  |  |  |  |  |  |  |  |  |  |  |  |
|  |  |  |  |  |  |  |  |  |  |  | 0.08 | -0.71 |  |  |  |  |  |  |  |  |  |  |  |  |  |  |
|  |  |  |  |  |  |  |  |  |  |  | 0.03 | -0.88 |  |  |  |  |  |  |  |  |  |  |  |  |  |  |
| Minimum |  | -0.65 |  | -0.69 |  |  |  |  |  | -0.38 |  | -0.88 |  |  |  |  |  |  |  |  |  | -0.54 |  |  |  |  |
| Q1 |  | -0.07 |  | -0.08 |  |  |  |  |  | 0.16 |  | -0.29 |  |  |  |  |  |  |  |  |  | -0.03 |  |  |  |  |
| Q2 |  | 0.79 |  | 0.82 |  |  |  |  |  | 0.97 |  | 0.59 |  |  |  |  |  |  |  |  |  | 0.74 |  |  |  |  |
| Q3 |  | 0.58 |  | 0.59 |  |  |  |  |  | 0.77 |  | 0.37 |  |  |  |  |  |  |  |  |  | 0.55 |  |  |  |  |
| Maximum |  | 1.66 |  | 1.73 |  |  |  |  |  | 1.78 |  | 1.47 |  |  |  |  |  |  |  |  |  | 1.51 |  |  |  |  |
| r | -0.74 | | -0.24 | | -0.06 | | -0.24 | | 0.25 | | -0.31 | | 0.85 | | -0.59 | | -0.33 | | 0.59 | | -0.28 | | 0.11 | | 0.19 | |
| Provenance | Coast | | Coast | | L Estuary | | Coast | | L Estuary | | Coast | | U Estuary | | Coast | | Coast | | U Estuary | | Coast | | L Estuary | | L Estuary | |
| Collection season | Spring | | Spring | | Winter | | Winter | | Summer | | Summer | | Winter | | Winter | | Winter | | Winter | | Autumn | | Winter | | Autumn | |
| **La Chora** | | | | | | | | | | | | | | | | | | | | | | | | | | |
|  | **1491** | | **1503** | | **1504** | | **1506** | | **1546** | | **1547** | | **1548** | | **1563** | | **1611** | | **1687** | | **1688** | |  |  |  |  |
|  | **δ^13^C_s_** | **δ^18^O_s_** | **δ^13^C_s_** | **δ^18^O_s_** | **δ^13^C_s_** | **δ^18^O_s_** | **δ^13^C_s_** | **δ^18^O_s_** | **δ^13^C_s_** | **δ^18^O_s_** | **δ^13^C_s_** | **δ^18^O_s_** | **δ^13^C_s_** | **δ^18^O_s_** | **δ^13^C_s_** | **δ^18^O_s_** | **δ^13^C_s_** | **δ^18^O_s_** | **δ^13^C_s_** | **δ^18^O_s_** | **δ^13^C_s_** | **δ^18^O_s_** |  |  |  |  |
| Shell edge | *-0.15* | *1.20* | *0.83* | *1.80* | *0.10* | *0.45* | *-0.62* | *0.22* | *-1.01* | *1.29* | *-0.33* | *1.48* | *-0.68* | *1.40* | *0.13* | *1.53* | *-0.59* | *1.68* | *-0.12* | *1.38* | *-0.09* | *1.38* |  |  |  |  |
|  | 0.06 | 0.93 | 1.02 | 0.79 | 0.02 | 0.69 | -0.62 | 0.07 | -0.81 | 1.52 | -0.31 | 1.47 | -0.24 | 1.44 | 0.10 | 1.39 | -0.62 | 1.64 | -0.02 | 1.24 | 0.01 | 1.65 |  |  |  |  |
|  | -0.50 | 0.40 | 1.33 | 1.78 | -0.09 | 0.20 | -0.74 | -0.32 | -0.81 | 1.49 | -0.07 | 1.53 | -0.67 | 0.65 | 0.09 | 0.69 | -0.48 | 0.73 | 0.03 | 1.30 | -0.09 | 1.10 |  |  |  |  |
|  | -0.45 | 0.41 | 1.49 | 1.41 | -0.25 | 0.13 | -1.03 | -0.16 | -0.52 | 1.58 | -0.01 | 1.65 | -0.55 | 0.74 | 0.15 | 1.44 | -0.55 | 0.62 | 0.09 | 1.22 | 0.26 | 1.26 |  |  |  |  |
|  | -0.66 | -0.23 | 1.48 | 0.95 | -0.43 | 0.39 | -1.29 | 0.74 | -0.71 | 1.05 | -0.18 | 1.54 | -0.63 | 0.40 | 0.27 | 1.38 | -0.55 | 0.10 | -0.01 | 0.91 | 0.15 | 1.01 |  |  |  |  |
|  | -0.28 | -0.01 | 1.38 | 0.95 | -0.49 | 0.31 | -1.21 | 0.83 | -0.72 | 1.43 | -0.03 | 1.49 | -0.55 | -0.29 | 0.32 | 1.64 | -0.67 | -0.03 | -0.10 | 0.84 | 0.09 | 0.80 |  |  |  |  |
|  | -0.36 | 0.47 | 0.83 | -0.16 | -0.17 | 0.42 | -1.03 | 0.94 | -0.92 | 0.95 | 0.29 | 1.62 | -0.22 | -0.30 | 0.32 | 1.66 | -0.35 | -0.13 | 0.04 | 0.76 | 0.16 | 0.53 |  |  |  |  |
|  | -0.46 | 0.59 | 0.48 | -0.40 | -0.05 | 0.07 | -0.60 | 1.41 | -0.66 | 1.25 | 0.36 | 1.57 | -0.49 | -0.11 | 0.51 | 1.78 | -0.14 | -0.11 | 0.08 | 0.82 | 0.16 | 0.88 |  |  |  |  |
|  | -0.68 | 0.82 | 0.53 | -0.48 | 0.00 | -0.22 | -0.03 | 1.47 | -0.47 | 1.08 | 0.07 | 1.54 | -0.37 | 0.04 | 0.53 | 1.74 | -0.19 | -0.19 | 0.23 | 0.61 | 0.09 | 0.72 |  |  |  |  |
|  | -0.43 | 1.04 | 0.30 | 0.19 | 0.35 | 0.29 | -0.09 | 1.43 | -0.72 | 0.94 | 0.24 | 1.67 | -0.50 | 0.08 | 0.55 | 1.68 | -0.02 | 0.29 | -0.03 | 0.49 | 0.16 | 0.55 |  |  |  |  |
|  | -0.01 | 1.26 |  |  |  |  |  |  |  |  | 0.13 | 1.59 | -0.63 | 0.62 |  |  |  |  |  |  | 0.22 | -0.01 |  |  |  |  |
|  | 0.52 | 1.58 |  |  |  |  |  |  |  |  | 0.04 | 1.34 | -0.96 | 0.73 |  |  |  |  |  |  | -0.04 | -0.25 |  |  |  |  |
|  | 0.57 | 1.68 |  |  |  |  |  |  |  |  | -0.13 | 1.06 | -1.30 | 1.00 |  |  |  |  |  |  | 0.03 | -0.46 |  |  |  |  |
|  | 0.46 | 1.97 |  |  |  |  |  |  |  |  | -0.53 | 0.97 | -0.26 | 1.93 |  |  |  |  |  |  | 0.12 | -0.73 |  |  |  |  |
|  | 0.50 | 1.57 |  |  |  |  |  |  |  |  | -0.45 | 0.90 | -0.16 | 1.51 |  |  |  |  |  |  | 0.27 | -0.12 |  |  |  |  |
|  | 0.15 | 0.97 |  |  |  |  |  |  |  |  | -0.25 | 0.94 | -0.13 | 1.26 |  |  |  |  |  |  | 0.41 | 0.19 |  |  |  |  |
|  | 0.60 | 0.94 |  |  |  |  |  |  |  |  | -0.14 | 0.92 | -0.14 | 0.87 |  |  |  |  |  |  | 0.47 | 0.61 |  |  |  |  |
|  | 0.82 | 1.44 |  |  |  |  |  |  |  |  | -0.04 | 1.03 | -0.56 | 0.49 |  |  |  |  |  |  | 0.27 | 0.54 |  |  |  |  |
|  | 0.61 | 1.07 |  |  |  |  |  |  |  |  | -0.10 | 0.79 | -0.66 | 0.12 |  |  |  |  |  |  | 0.40 | 0.72 |  |  |  |  |
|  | 0.32 | 0.95 |  |  |  |  |  |  |  |  | -0.06 | 0.97 | -0.11 | 0.65 |  |  |  |  |  |  | 0.39 | 0.86 |  |  |  |  |
|  | 0.22 | 0.55 |  |  |  |  |  |  |  |  | -0.27 | 0.66 | -0.05 | 0.50 |  |  |  |  |  |  | -0.02 | 0.64 |  |  |  |  |
|  | 0.32 | 0.41 |  |  |  |  |  |  |  |  | -0.23 | 0.50 | -0.07 | 0.07 |  |  |  |  |  |  | -0.48 | 0.52 |  |  |  |  |
|  | 0.28 | 0.85 |  |  |  |  |  |  |  |  | -0.17 | 0.75 | -0.01 | 0.09 |  |  |  |  |  |  | -0.31 | 0.46 |  |  |  |  |
|  | -0.03 | 0.69 |  |  |  |  |  |  |  |  | -0.05 | 0.83 | 0.30 | 0.18 |  |  |  |  |  |  | -0.27 | 0.59 |  |  |  |  |
|  | 0.17 | 0.83 |  |  |  |  |  |  |  |  | -0.21 | 0.72 | 0.56 | -0.10 |  |  |  |  |  |  | -0.14 | 1.30 |  |  |  |  |
|  | 0.20 | 0.62 |  |  |  |  |  |  |  |  | -0.48 | 0.44 | 0.59 | -0.08 |  |  |  |  |  |  | -0.69 | 0.32 |  |  |  |  |
|  | 0.24 | 0.54 |  |  |  |  |  |  |  |  | -0.49 | 0.10 | 0.62 | -0.39 |  |  |  |  |  |  | -0.14 | 1.29 |  |  |  |  |
|  | 0.24 | 0.44 |  |  |  |  |  |  |  |  | -0.73 | -0.34 | 0.29 | -0.39 |  |  |  |  |  |  | -0.19 | 1.42 |  |  |  |  |
|  | -0.14 | 0.02 |  |  |  |  |  |  |  |  | -0.64 | -0.23 | 0.29 | -0.23 |  |  |  |  |  |  | 0.05 | 1.35 |  |  |  |  |
|  | 0.04 | -0.05 |  |  |  |  |  |  |  |  | -0.12 | -0.03 | 0.53 | 0.09 |  |  |  |  |  |  | -0.11 | 1.30 |  |  |  |  |
| Minimum |  | -0.23 |  |  |  |  |  |  |  |  |  | -0.34 |  | -0.39 |  |  |  |  |  |  |  | -0.73 |  |  |  |  |
| Q1 |  | 0.32 |  |  |  |  |  |  |  |  |  | 0.17 |  | 0.19 |  |  |  |  |  |  |  | -0.14 |  |  |  |  |
| Q2 |  | 1.14 |  |  |  |  |  |  |  |  |  | 0.92 |  | 1.06 |  |  |  |  |  |  |  | 0.75 |  |  |  |  |
| Q3 |  | 0.94 |  |  |  |  |  |  |  |  |  | 0.73 |  | 0.84 |  |  |  |  |  |  |  | 0.53 |  |  |  |  |
| Maximum |  | 1.97 |  |  |  |  |  |  |  |  |  | 1.67 |  | 1.93 |  |  |  |  |  |  |  | 1.65 |  |  |  |  |
| r | 0.59 | | 0.70 | | -0.02 | | 0.42 | | 0.07 | | 0.72 | | -0.41 | | 0.69 | | -0.51 | | -0.35 | | -0.11 | |  |  |  |  |
| Provenance | U Estuary | | U Estuary | | L Estuary | | U Estuary | | L Estuary | | U Estuary | | Coast | | U Estuary | | Coast | | Coast | | Unknown | |  | |  | |
| Collection season | Autumn | | Winter | | Spring | | Autumn | | Winter | | Winter | | Winter | | Winter | | Winter | | Winter | | Winter | |  |  |  |  |

Figure S1. Archaeological δ^13^C - δ^18^O correlation coefficients in relation to the modern counterparts previously published by Milano et al. (2020). The highlighted areas represent the 95% confidence intervals of the modern correlation coefficients for the three habitats considered.


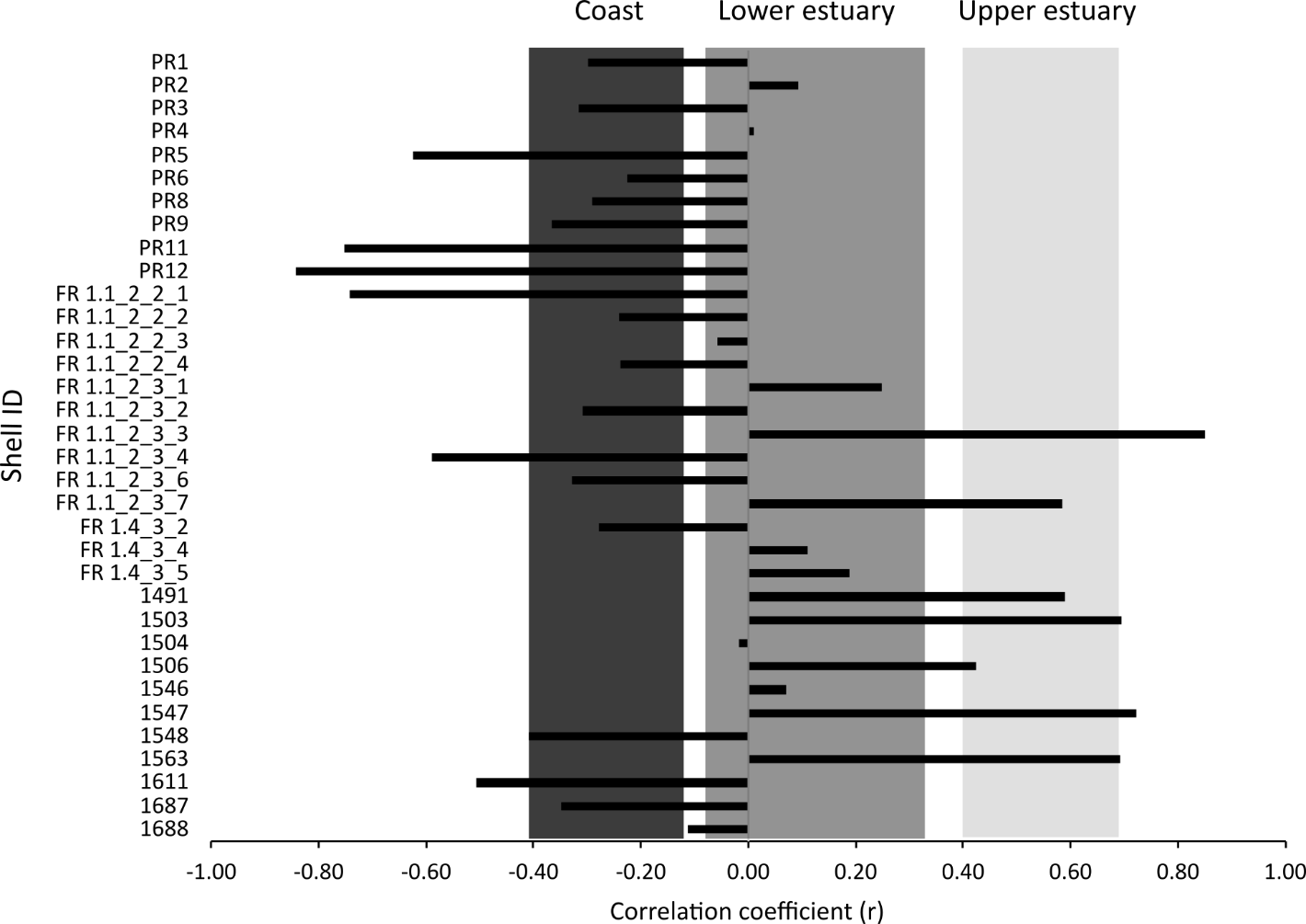


**Description of the sites**

**Peña del Perro rock-shelter**

The rock shelter of the Peña del Perro (usually abbreviated as El Perro) is located in Santoña (Cantabria), specifically on the SE slope of Monte Buciero. It is situated in an area that dominates the Bay of Laredo, although in the early Holocene the landscape would have been very different, with the sea level lower than at present, and therefore, with the coastline several kilometres further on North than today. Thus, the rock shelter would have been located at that time dominating a valley area. Today, the connection between this valley and the rock shelter has been dismantled by the sea erosive action on the lower slopes of Monte Buciero, as occurs in the nearby Cave of La Fragua.

The rock shelter has a triangular ground plan, a dozen meters wide and deep, with opening faces on the southeast side (Fig. S2). Along the west wall there was a cemented shell midden, whose remains reached almost two meters above the current surface (González Morales and Díaz Casado, 1992, 2000).


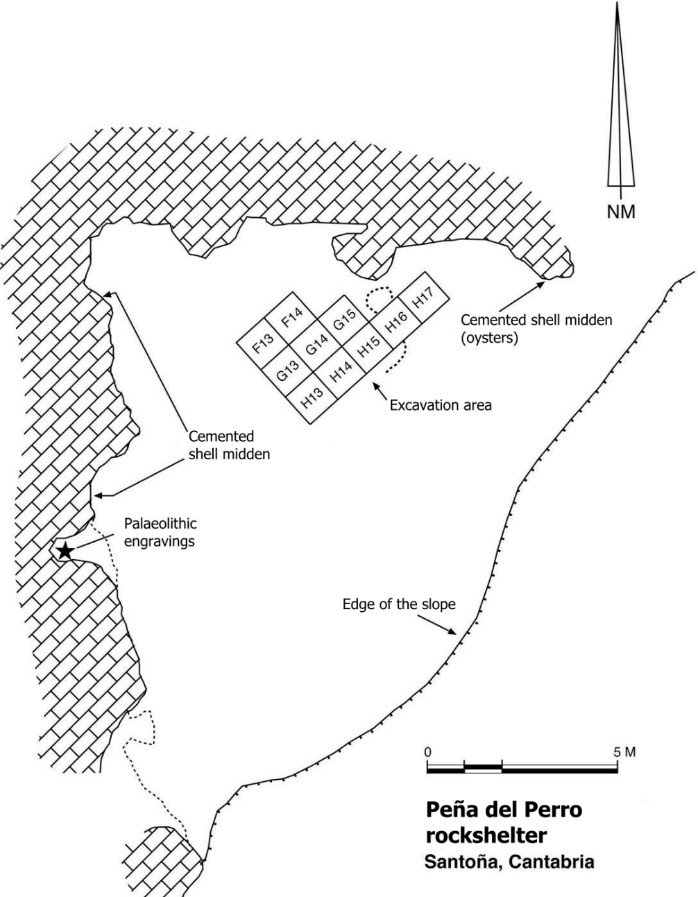


Fig. S2: Plan of Peña del Perro rock shelter and the excavated areas.

The site was excavated between 1985 and 1990 under the direction of M.R González Morales and Y. Díaz Casado (González Morales and Díaz Casado, 1992, 2000). The excavation allowed the identification of three chronocultural periods, one corresponding to the Mesolithic (Level 1), another to the Azilian (Level 2a/b), and a third from the Magdalenian period (Level 2c).

The stratigraphic sequence of the deposit corresponding to the Mesolithic is limited to level 1 (González Morales and Díaz Casado, 1992, 2000). This level showed a relative complexity in its internal arrangement, due to the fact that it was formed by the accumulation of shells, charcoal and the remains of unstructured hearths that partially overlap throughout the sequence. This type of arrangement is common in shell deposits of a certain volume, which are developed on a limited surface, as in this case. This complexity led the excavators to divide the level into five sublevels (1.1 to 1.5). However, due to lateral variations, these sublevels did not appear on the entire excavated surface.


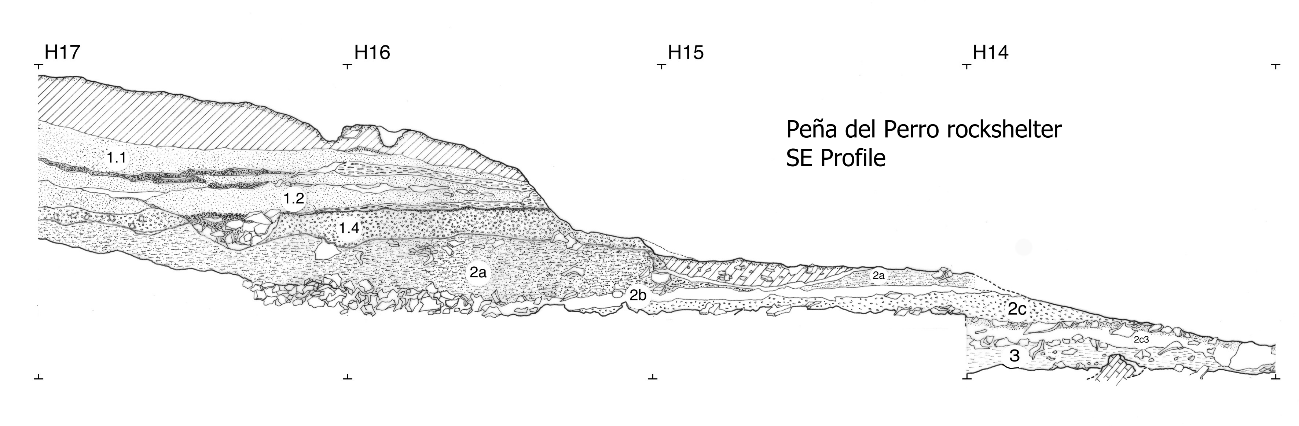


Fig. S3: Stratigraphy of the southeast profile at Peña del Perro rock shelter.

The *Mytilus galloprovincialis* shells used in this work come from stratigraphic unit 1.5, excavated in square H16 (sectors 2, 6, 7 and 9). The dating of units 1.5, 1.4 and 1.3 place the sequence at the very start of the Mesolithic in the region (Table S3).

Table S3: Radiocarbon dates from Mesolithic units at El Perro.

| **Unit** | **Square** | **Material** | **Taxa** | **Date BP** | **Interval cal BP** | | **Lab Ref.** | **Method** |
| --- | --- | --- | --- | --- | --- | --- | --- | --- |
| 1.3 | H16 | Charcoal |  | 9260±110 | 10725 | 10225 | GrN-18116 | C14 |
| 1.4 | H16 | Shell | *P. vulgata* | 9660±45 | 11059 | 10588 | ﻿OxA-33179 | ^14^C AMS |
| 1.4 | H16 | Shell | *P. vulgata* | 9780±40 | 11173 | 10758 | OxA-34388 | ^14^C AMS |
| 1.4 | H16 | Shell | *P. vulgata* | 9770±40 | 11165 | 10747 | OxA-34389 | ^14^C AMS |
| 1.5 | H16 | Shell | *M. galloprovincialis* | 9690±30 | 11074 | 10653 | ICA-19S/10106 | ^14^C AMS |
| 1.5 | H16 | Shell | *M. galloprovincialis* | 9910±40 | 11340 | 10953 | ICA-19S/10105 | ^14^C AMS |

**La Fragua cave**

La Fragua cave is located in the town of Santoña (Cantabria, Spain), and more specifically on the southeast slope of Monte Buciero, about 800 m northeast of the Peña del Perro rock shelter. Its UTM coordinates are x: 465425 y: 4810405 and its altitude above sea level is approximately 130 m. It is a small cavity, barely three meters deep by almost 10 m in its widest part, and it is oriented to the east (Fig. S4).

The first archaeological works at the site were developed between 1990 and 1996 under the direction of Manuel R. González Morales (González Morales, 2000). During this period, an area of ​​six squared meters was excavated. The stratigraphic sequence uncovered during the excavation revealed the existence of a Mesolithic shell midden (Level 1, divided into lower, mid and upper), an Azilian level (Level 3) and a level corresponding to the Upper Magdalenian (Level 4). In 2015 a new archaeological intervention was carried out under the direction of Igor Gutiérrez-Zugasti, to more precisely characterize the site stratigraphy, and especially the Mesolithic shell midden (Fig. S5). The excavation of Mesolithic units was carried out over an area of ​​33.3 x 66.6 cm in square A3 (sectors 2 and 3).


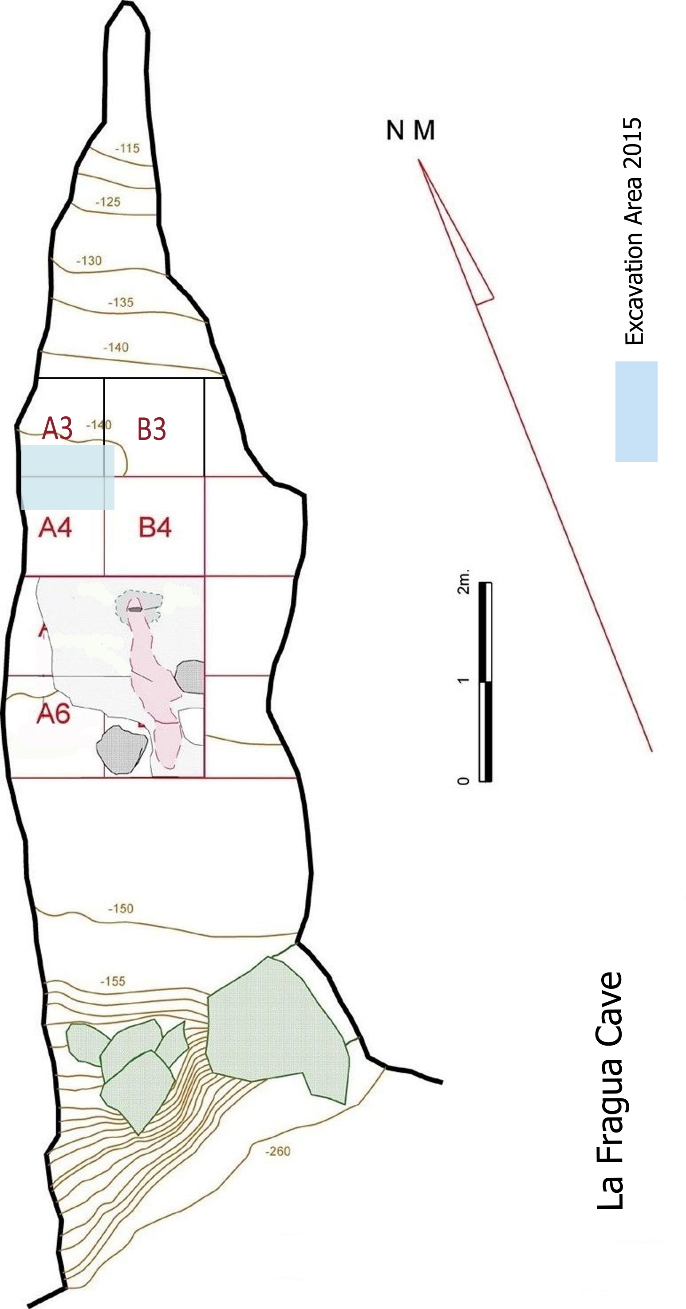


Fig.S4: Plan of La Fragua cave and the excavated areas.

During the 2015 archaeological intervention, a series of stratigraphic units (SU) were identified, which are described below:

SU 0: Light brown sediment, compacted by trampling. It is disturbed but it includes archaeological material (mainly shells and some bones).

SU 1.1: Very loose greyish-brown sediment with abundant quantity of shells, some bones and charcoal.

SU 1.2: Ash grey sediment with archaeological material, mainly shells. It is an ash unit with some charcoal remains.

SU 1.3: Very defined carbonaceous line, located in sector 3. And composed mainly of charcoal.

SU 1.4: Loose sediment with various colorations (reddish, blackish, brown/grey). This unit includes different events related to fire making. However, a distinction of the individual fire events was not possible. Mollusc shells are the most abundant archaeological material.

SU 1.5: Loose grey/white sediment. It is an ash unit limited exclusively to the eastern area of ​​sector 3. There is hardly any archaeological material.

SU 1.6: Loose reddish sediment, with blackish areas due to the presence of charcoal. It is limited to sector 3. Although shells appear, their abundance is low.

SU 3: Loose orange/yellow sediment, with inclusions of crust and gravel. Charcoal, mammalian bones and lithics appear. The presence of burrows is remarkable, especially in the lower part of the unit. This indicates intense activity by animals that removed part of the original sediment and filled it with shell remains of the overlying units and materials similar to the ones documented *in situ* in this same unit. This unit was considered as Azilian during the González-Morales excavation. However, new radiocarbon dates from the 2015 excavation place the formation of this unit during the Mesolithic.


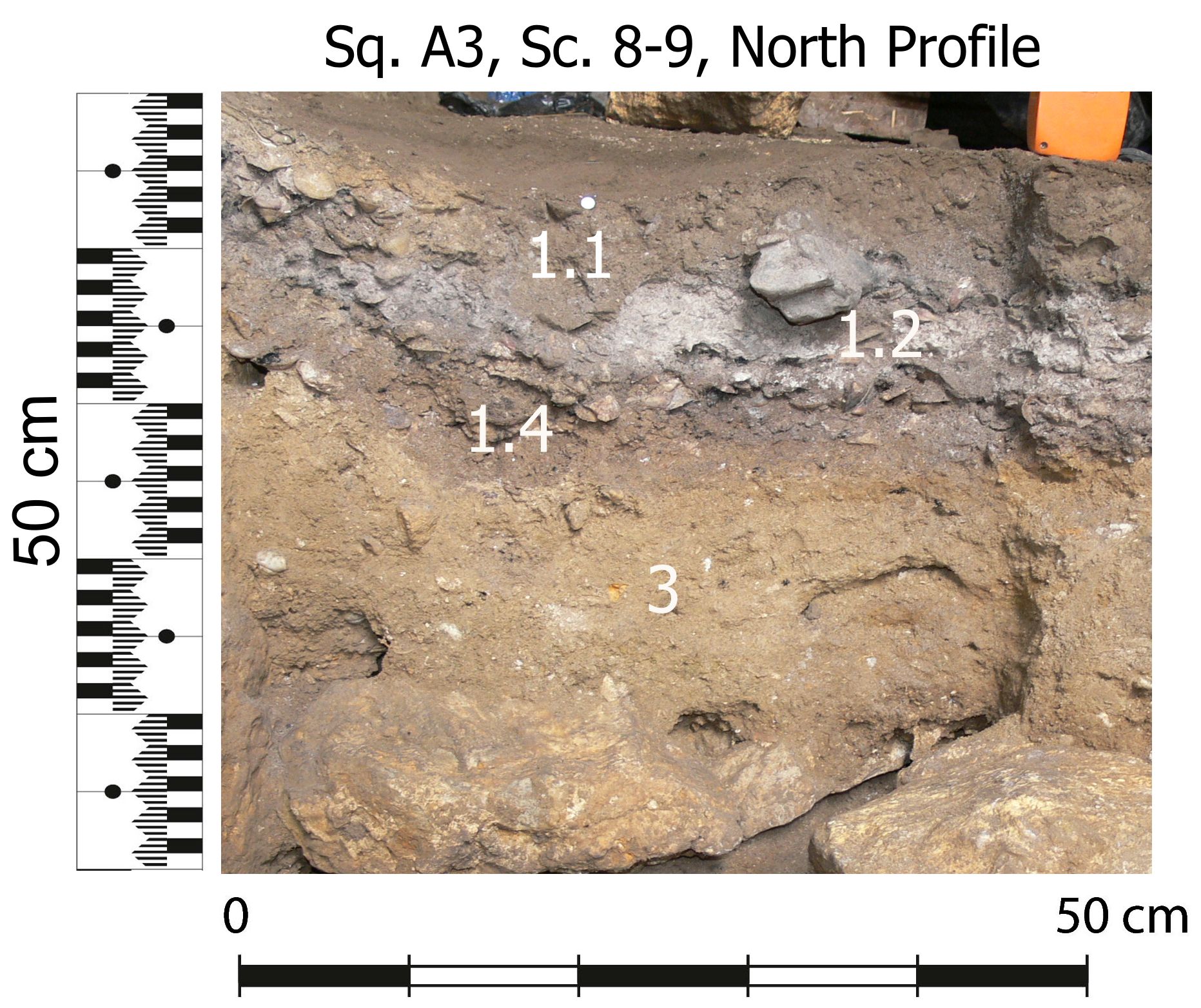


Fig. S5: Stratigraphic profile from La Fragua cave showing Mesolithic units during the 2015 excavation.

The *Mytilus galloprovincialis* shells used in this work come from stratigraphic units 1.1 and 1.4 excavated in the square A3 (sectors 2 and 3). Radiocarbon dates place the sequence during the Mesolithic (Table S4).

Table S4: Radiocarbon dates from Mesolithic units at La Fragua.

| **Unit** | **Square** | **Material** | **Taxa** | **Date BP** | **Interval cal BP** | | **Lab Ref.** | **Method** |
| --- | --- | --- | --- | --- | --- | --- | --- | --- |
| 1 Sup | A4 | Charcoal | - | 6650±120 | 7735 | 7315 | GrN-20963 | C14 |
| 1 Mid | A4 | Charcoal | - | 6860±60 | 7833 | 7582 | GrN-20964 | C14 |
| 1 Low | A4 | Charcoal | - | 7530±70 | 8446 | 8182 | GrN-20665 | C14 |
| 1.1 | A3 | Shell | *M. galloprovincialis* | 7320±40 | 8030 | 7710 | ICA-17S/0436 | ^14^C AMS |
| 1.4 | A3 | Shell | *M. galloprovincialis* | 7500±40 | 8249 | 7916 | ICA-17S/0435 | ^14^C AMS |
| 3 | A3 | Bone | *Cervus elaphus* | 8940±40 | 10208 | 9908 | ICA-17B/0434 | ^14^C AMS |
| 3 | A4 | Charcoal | *-* | 9600±140 | 11255 | 10517 | GrN-20966 | C14 |

**La Chora cave**

La Chora cave is located in the town of San Pantaleón de Aras, in the municipality of Voto (Cantabria). The cave is situated at the base of a small hill next to a meadow that serves as a drain (Fig. 1). It is located very close to the Clarín river and ca 4 km from the Asón river upper estuary. It is oriented to the southeast and is at the base level of the Aras valley, its UTM coordinates are x: 459383 y: 4810405, and its elevation with respect to sea level is about 40 m (Fig. S5). The cavity is small. It has two small entrances (one of them is almost closed at present), which lead to a lobby of barely 15 m wide by four metres deep. In addition to the hall, the cave has a small gallery of about 6 m wide and 15 m long. La Chora was excavated in 1962 under the direction of Joaquín González Echegaray and Miguel Ángel García Guinea (González Echegaray et al., 1963). Although they identified the remains of a shell midden in the inner gallery, the excavation focused mainly on ​​the entrance area (Fig. 1), where they uncovered Upper Magdalenian deposits. Later, González Sainz (1989) and Yudego Arce (1995) reviewed the materials from González Echegaray's excavation. They concluded that at least levels I, II and III (and probably IV) could not be framed within of the Magdalenian, since they presented characteristics more typical of the Azilian. Yudego Arce's review of the site also culminated in taking samples of the interior shell midden to characterize it and to approximate its chronology. A charcoal sample was dated 6360 ± 80 BP, indicating the existence of a Mesolithic occupation of the cave (González Morales et al., 2000).

In 2017, a new archaeological intervention was carried out at the site under the direction of Igor Gutiérrez Zugasti (Fig. S6, S7). The objective of this intervention was to characterize the stratigraphy of the inner gallery and determine the degree of integrity of the Mesolithic shell midden. The excavation was carried out over an area of ​​1 x 0.50 m in square M97 (sectors C and D).


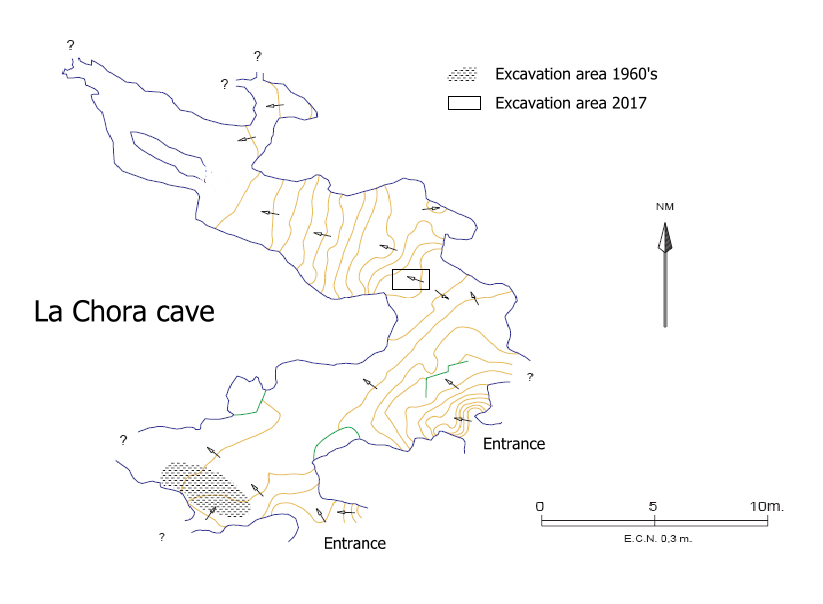


Fig. S6: Plan of La Chora cave with the location of the González Echegaray excavation (entrance) and the sampling carried out in 2017 (interior gallery).

During the excavation, a series of stratigraphic units were identified, which are described below:

SU 101A: Compact brown clayey and compact sediment, with archaeological materials (mainly shells), but with evidence of disturbance. Isolated remains of a cemented shell midden appeared in secondary position. This unit continues below the EU 101B (crust), with disturbed sediments due to the presence of a burrow.

SU 101B: Stalagmitic crust, formed in the area of ​​the slope sealing the underlying levels, especially in the northern area of ​​the square.

SU 102: Compact orange-brown clay, with archaeological materials, mainly shells. It is the first intact unit in the sequence.

SU 103: Shell midden unit, with compact orange-brown clay, containing abundant archaeological remains, especially shells and bones.

SU 104: Shell midden unit with compact orange-brown clay sediment, containing less archaeological remains than the previous unit.

SU 105: Loose, brown and sandy sediment, with little archaeological material.

SU 106: Fine layer of loose, yellowish and sandy sediment, situated between the 105 and 107 SUs. Almost sterile from the archaeological point of view.

SU 107: Dark brown, sandy and relatively loose sediment, with little archaeological material.

SU 108: Shell midden unit, with brown clayed sediment and abundant archaeological remains (shells, bones, lithic…).


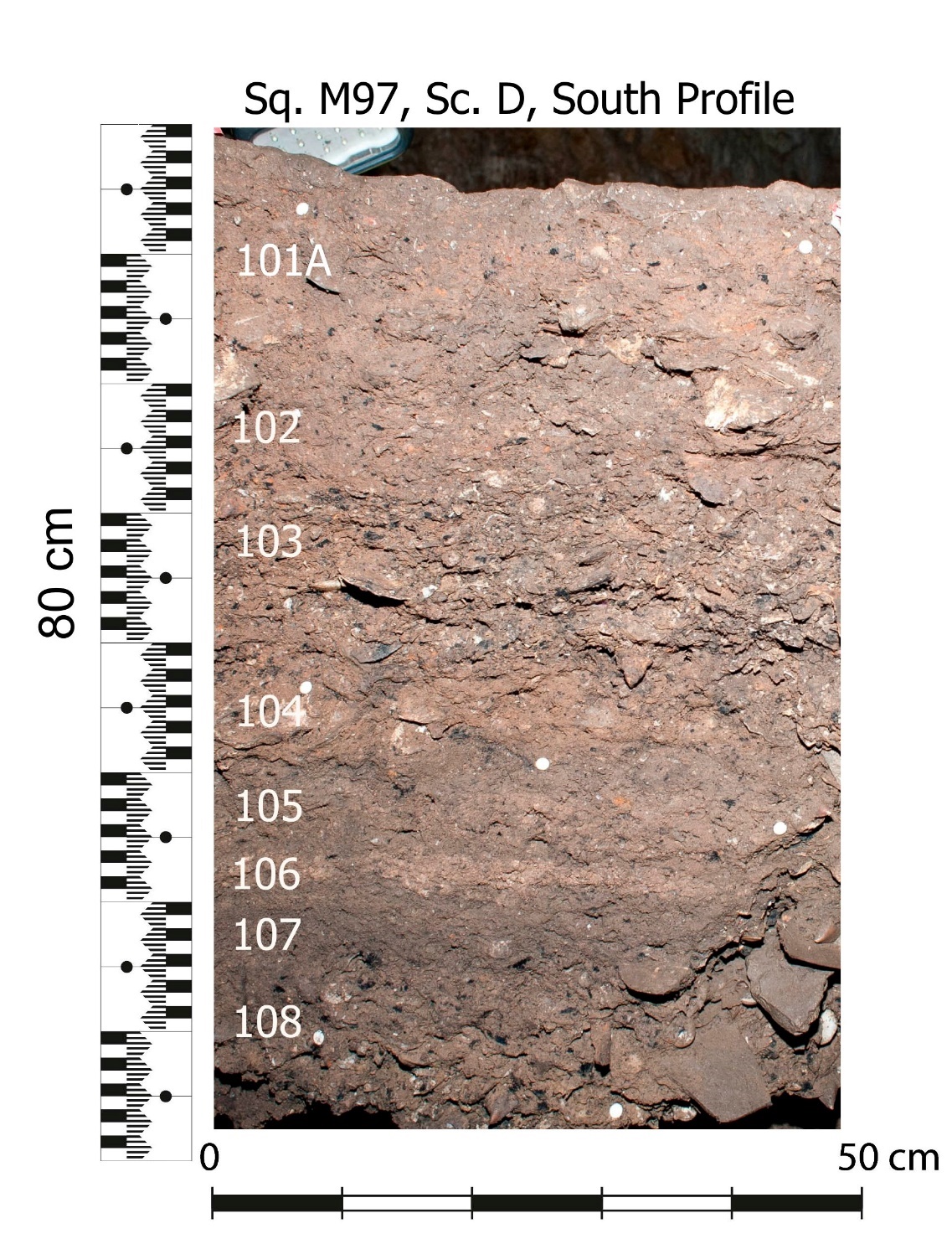


Fig. S7: Stratigraphic profile from La Chora cave showing Mesolithic units after the 2015 excavation.

The *Mytilus galloprovincialis* shells used in this work come from stratigraphic units 103 and 104 excavated in square M97, sector D. The dating of units 103, 104 and 108 placed the complete sequence in the Mesolithic (Table S5).

Table S5: Radiocarbon dates from Mesolithic units at La Chora.

| **Unit** | **Square** | **Material** | **Taxa** | **Date BP** | **Interval cal BP** | | **Lab Ref.** | **Method** |
| --- | --- | --- | --- | --- | --- | --- | --- | --- |
| - | - | Charcoal |  | 6360±80 | 7429 | 7027 | GrN-20961 | ^14^C |
| 103 | M97 | Shell | *M. galloprovincialis* | 7390±40 | 8136 | 7796 | ICA-19S/0177 | ^14^C AMS |
| 104 | M97 | Shell | *M. galloprovincialis* | 7430±40 | 8166 | 7845 | ICA-19S/0176 | ^14^C AMS |
| 108 | M97 | Shell | *M. galloprovincialis* | 9050±40 | 10177 | 9759 | ICA-19S/0175 | ^14^C AMS |

**References**

González Echegaray, J., García Guinea, M.A., Begines Ramirez, A. Cueva de La Chora, (Dirección General de Bellas Artes, Madrid, 1963).

González Morales, M.R. La Prehistoria de las Marismas: Excavaciones en la Cueva de La Fragua (Santoña). Campañas de 1990, 1991, 1993, 1994 y 1996. In: Actuaciones arqueológicas en Cantabria, 1984-1999, Gobierno de Cantabria, Santander, Ontañón, R. (Ed.), 177-179 (2000).

González Morales, M.R., Díaz Casado, Y. Excavaciones en los abrigos de la Peña del Perro (Santoña, Cantabria). Estratigrafía, cronología y comentario preliminar de sus industrias. *Veleia* **8-9**, 43-64 (1992).

González Morales, M.R., Díaz Casado, Y. La Prehistoria de las Marismas: excavaciones arqueológicas en los abrigos de la Peña del Perro (Santoña). In: Actuaciones arqueológicas en Cantabria, 1984-1999, Gobierno de Cantabria, Santander, Ontañón, R. (Ed.), 93-96 (2000).

González Morales, M.R., Yudego, C., Ituarte, C. La Prehistoria de las Marismas: Prospección arqueológica de la zona del bajo Asón y marismas de Santoña y toma de muestras en los yacimientos de las cuevas del Otero, La Chora y El Valle. In: Actuaciones Arqueológicas en Cantabria 1984-1999, Gobierno de Cantabria, Santander, Ontañón, R. (Ed.) 151-154 (2000)

González Sainz, C., El Magdaleniense Superior-Final en la región cantábrica, Tantín-Universidad de Cantabria, Santander (1989).

Yudego Arce, C. Estudio arqueológico de las ocupaciones post-magdalenienses de los yacimientos de la cuenca baja del Asón y valle de Aras (Cuevas de La Chora, El Otero, El Valle y Cobrante), Trabajo de Investigación de Tercer Ciclo, Universidad de Cantabria, Santander (1995).
